# rBCG-LTAK63 enhances protection against tuberculosis by inducing autophagy and circadian gene regulation

**DOI:** 10.3389/fimmu.2025.1695560

**Published:** 2025-10-24

**Authors:** Lázaro M. Marques-Neto, Monalisa M. Trentini, Ana Carolina R. Moreno, Silas F. Eto, Ana Carolina O. Carvalho, Almiro P.S. Neto, Murilo S. Amaral, André G. C. Martins, André N. A. Gonçalves, Ana Marisa Chudzinski-Tavassi, Alex I. Kanno, Aldo Tagliabue, Diana Boraschi, Sergio Verjovski-Almeida, Helder Nakaya, Leonardo P. Farias, Pablo I. P. Ramos, Luciana C. C. Leite

**Affiliations:** ^1^ Laboratório de Desenvolvimento de Vacinas, Instituto Butantan, São Paulo, Brazil; ^2^ Center of Excellence in New Target Discovery (CENTD), Instituto Butantan, São Paulo, Brazil; ^3^ Laboratório de Medicina e Saúde Pública de Precisão (MeSP2), Instituto Gonçalo Moniz, Fundação Oswaldo Cruz (FIOCRUZ-BA), Salvador, Brazil; ^4^ Laboratório de Ciclo Celular, Instituto Butantan, São Paulo, Brazil; ^5^ Department of Clinical and Toxicological Analyses, School of Pharmaceutical Sciences. University of São Paulo, São Paulo, Brazil; ^6^ Micromanufacturing Laboratory, Institute for Technological Research, São Paulo, Brazil; ^7^ Hospital Israelita Albert Einstein, São Paulo, Brazil; ^8^ Shenzhen Institutes of Advanced Technology (SIAT), Shenzhen-Guangdong, China; ^9^ Shenzhen University of Advanced Technology (SUAT), Shenzhen-Guangdong, China; ^10^ Instituto de Química, Departamento de Bioquímica, Universidade de São Paulo, São Paulo, Brazil; ^11^ Institut Pasteur de São Paulo, São Paulo, Brazil; ^12^ Centro de Integração de Dados e Conhecimentos para Saúde (CIDACS), Instituto Gonçalo Moniz, Fundação Oswaldo Cruz (FIOCRUZ-BA), Salvador, Brazil

**Keywords:** tuberculosis, recombinant BCG, transcriptome, adjuvant, vaccine

## Abstract

Tuberculosis (TB) remains a global public health challenge, with the current BCG vaccine providing limited efficacy in adults, and available treatments being lengthy and debilitating. To overcome these challenges, we have previously developed a recombinant BCG strain expressing the detoxified *E. coli* Heat-Labile Toxin (LTAK63), providing increased protection in mouse models and reduced lung pathology. Here, using systems biology and RNA sequencing of lung tissues in a murine model, we uncover the molecular mechanisms underlying rBCG-LTAK63’s increased protection. Immunization triggered early activation of cAMP-related pathways, leading to hypoxia, autophagy, and circadian rhythm gene regulation. These processes were associated with an enhanced innate immunity and promoted long-lasting Th1/Th17 adaptive responses. Upon challenge, mice immunized with rBCG-LTAK63 exhibited an earlier onset of interferon-gamma response, reduced bacterial burden, and improved lung histopathology. Notably, circadian rhythm regulation was directly linked to a controlled inflammatory response and reduced migration of infection-susceptible cells, resulting in decreased immunopathology. Our findings demonstrate that rBCG-LTAK63 orchestrates protection through the integration of metabolic and temporal immune pathways. This work provides mechanistic insights into how rational vaccine design can reprogram host immunity to enhance protection and reduce pathology, supporting rBCG-LTAK63 as a promising next-generation TB vaccine candidate.

## 1 Introduction

Tuberculosis (TB) imposes a major global health impact, resulting in over ten million cases and 1.3 million fatalities per year worldwide (1). The Bacillus Calmette-Guérin (BCG) vaccine, although protective in infants, shows variable efficacy in preventing TB, especially in adults and in regions with a high burden of HIV/AIDS, where pulmonary TB is prevalent (2–4). Given these constraints, there is a pressing demand for a more effective and long-lasting TB vaccine, which would be essential to decrease the worldwide impact of the disease.

*Mycobacterium tuberculosis* (*Mtb*), the main causative agent of TB in humans, invades host macrophages and employs diverse immune evasion strategies. Protection against TB primarily relies on coordinated Th1, Th17 and CD8 immune responses, with the contribution of B cell responses and innate immunity. Maintaining a balance between these immune responses is crucial; the absence of such responses results in uncontrolled bacterial replication, while their excess can lead to severe disease, tissue injury, and cell exhaustion (5, 6). Previous studies, using both mouse TB models and human TB cohorts, have shown that, as the infection progresses, an imbalance occurs between type I and type II interferon (IFN) responses, favoring a type I response. This imbalance results in neutrophil over-recruitment and activation, along with a decline in B cells, natural killer (NK) cells, and T cell effector responses (7).

In the development of novel TB vaccines, researchers have historically focused on key protection-related biomarkers, aiming to induce specific T cell populations, particularly central memory, and tissue-resident cells (8, 9). Memory-like innate responses are also important, as well as induction of mechanisms such as apoptosis and phagolysosome escape (as in VPM1002); BCG-induced autophagy has also been pursued in attempts to prevent the disease (5). The most advanced strategies include either immune regulation-based approaches, such as in VPM1002 (10, 11), or antigen-based strategies, such as in MTBVAC (12, 13). These promising vaccines are in Phase II/III clinical trials.

On the other hand, it has been shown that intravenous (i.v.) administration of BCG at a tenfold higher dose than standard intradermal administration can induce robust immune responses and confer high levels of protection against *Mtb* in mouse and macaque models (14). While these findings offer valuable insights into protective immune mechanisms, immunization led to marked enlargement of lymph nodes and spleens in animal models, raising safety concerns. Furthermore, there are significant logistical challenges in delivering such high vaccine doses i.v. in large-scale immunization campaigns, particularly in low- and middle-income countries (LMICs), rendering this strategy unsuitable where it is most needed.

More recently, the recombinant fusion protein (M72) in combination with a strong adjuvant (ASO1_E_) showed ~50% reduction in activation of latent TB infection in a Phase IIb trail. These results evoked high expectations leading to the initiation of Phase III clinical trials (15). However, there are concerns as to availability of the adjuvant, as the QS-21 bark extract from the tree *Quillaja saponaria* used in combination with monophosphoryl lipid A, can face supply limitations (16).

On a whole, although there are a variety of vaccine strategies in different stages of developments, there is still a need for improved, scalable, and safe TB vaccine strategies. We investigated an alternative strategy, modulating the immune response to BCG by expression of a strong adjuvant (17). Vaccine adjuvants may modulate immune responses by engaging otherwise unaddressed immune pathways. The *E. coli* Heat Labile Toxin (LT) is a very potent toxin that has a variety of well-known adjuvant properties (17). Genetically detoxified versions of LT, such as LTK63, LTK72 and double mutant LT (dmLT), have been developed for use as mucosal vaccine adjuvants. It is considered that LTK72 and dmLT have no toxic activity, while LTK63 has very low residual toxicity (17, 18). However, recent clinical trials using LTK63 as an intranasal vaccine adjuvant were interrupted due to the induction of transient Bells’ Palsy paralysis (19). This effect is attributed to the GM1 binding properties of the LTB subunit pentamer, which leads to internalization of the active LTA subunit. The LTA subunit drives activation of cytosolic ADP-ribosylation factors, intoxicating cells through cAMP overproduction. No toxic properties have been reported for the modified LTAK63, which is not expected to have potential adverse effects as it lacks the LTB subunit (20).

On these grounds, we have previously constructed a recombinant BCG (rBCG) strain expressing LTAK63 as a TB vaccine (21). Immunized mice showed an increased Th1/Th17 response, resulting in a 2-log reduction in lung bacteria upon subsequent *Mtb* challenge. The intratracheal *Mtb* infection following immunization induced immunomodulatory responses in the lungs of infected animals, characterized by increased TGF-β and decreased Th1 and Th17 cytokine production, together with a marked decrease of immunopathology in the lung (21). rBCG-LTAK63 immunization also induced an increase in single and polyfunctional effector and central memory cells as compared with wild type BCG, providing superior protection against *Mtb* challenge even after 180 days post immunization in an intranasal *Mtb* challenge model (22). Further work produced a vaccine suitable for human use, through a complemented auxotrophic strategy. Using CRISPR/cas9, the BCGΔ*lysA* strain was generated, to knock out *lysA*, which was then complemented with a mycobacterial expression vector carrying *ltak63* and *lysA* genes (23). This strain exhibited the same protective efficacy as previously demonstrated in an *Mtb* intranasal challenge model (23). The investigation of the mechanisms involved in the improved protection induced by this strain can uncover novel biomarkers of protection or the promotion of new vaccination strategies.

Pioneering work by Querec et al. and Nakaya et al. established the framework for systems vaccinology (24, 25). Using advanced techniques and new technological platforms, these studies enabled the measurement and characterization of extensive gene networks. Computational and statistical tools were employed to manage complex data, yielding insights into the molecular signatures of yellow fever and influenza vaccines, and uncovering novel immune-mediated mechanisms of vaccine responses (24, 25). Systems biology has since been applied across several viral, bacterial, and parasite vaccines with diverse presentation systems, to identify gene expression signatures distinguishing protected from unprotected individuals. Notably, each vaccine class is linked to the induction of specific signatures (26).

We used RNA sequencing (RNA-seq) focused on the lungs, the main site of tuberculosis disease, at various time points before and after *Mtb* exposure, to comprehensively study the impact of rBCG-LTAK63 vaccination in mice, to identify gene signatures aiming to understand how these alterations influence lung immune responses and contribute to protection against TB. The results highlight a potential role for cAMP induction, leading to activation of stress responses, autophagy and circadian rhythm pathways, accelerating IFN-γ responses and regulating inflammatory responses after challenge, which improve protection against tuberculosis.

## 2 Materials and methods

### 2.1 Experimental animals and ethics approval

This study adheres to the Guide for the Care and Use of Laboratory Animals, following the guidelines set by the Committee of SBCAL (Sociedade Brasileira de Ciência em Animais de Laboratório) and received approval from the Animal Research Ethical Committee of Instituto Butantan (Number: 3435250619). Pathogen-free female BALB/c mice, aged 4–8 weeks, were housed in ABSL-2 racks equipped with HEPA-filtered air intake and exhaust systems at the Butantan Institute Animal Facility. Animals were accommodated in the Laboratório de Desenvolvimento de Vacinas animal care facility and provided with ad libitum access to water and food. Environmental conditions were controlled to maintain a temperature range of 20–24°C, relative humidity between 40–70%, and a 12-h light/dark cycle.

### 2.2 Bacterial strains, media, and growth conditions

The rBCG-LTAK63 strain used in this study, which is auxotrophic/complemented (*ΔlysA::lysA-ltak63*), was generated using the BCG Danish strain as described by Moraes et al. (23). Wild-type BCG Danish was used in the control group. The BCG strains were grown in Middlebrook 7H9 medium (Difco, Detroit, MI, USA) supplemented with 10% OADC (oleic acid, albumin-dextrose-catalase; BBL, Cockeysville, MD, USA), 0.5% glycerol, and 0.05% Tween 80 (MB7H9). For quantifying colony-forming units (CFUs), the strains were cultivated on Middlebrook 7H10 agar (Difco) supplemented with 10% OADC (BBL) (MB7H10). *Mycobacterium tuberculosis* (H37Rv) was also cultivated in MB7H9 medium. When the bacterial culture reached an O.D (600 nm) of 0.4 – 0.6, the bacteria were centrifuged (5000 × g, 15min, 4°C), and the pellet resuspended in 10% glycerol and stored at a temperature of -80°C until use. The CFU of BCG, rBCG-LTAK63, and *Mtb* were determined by plating serial dilutions in MB7H10. The bacteria were incubated at 37°C with 5% CO_2_ for 14–21 days.

### 2.3 Immunization and experimental design

**Figure 1:**
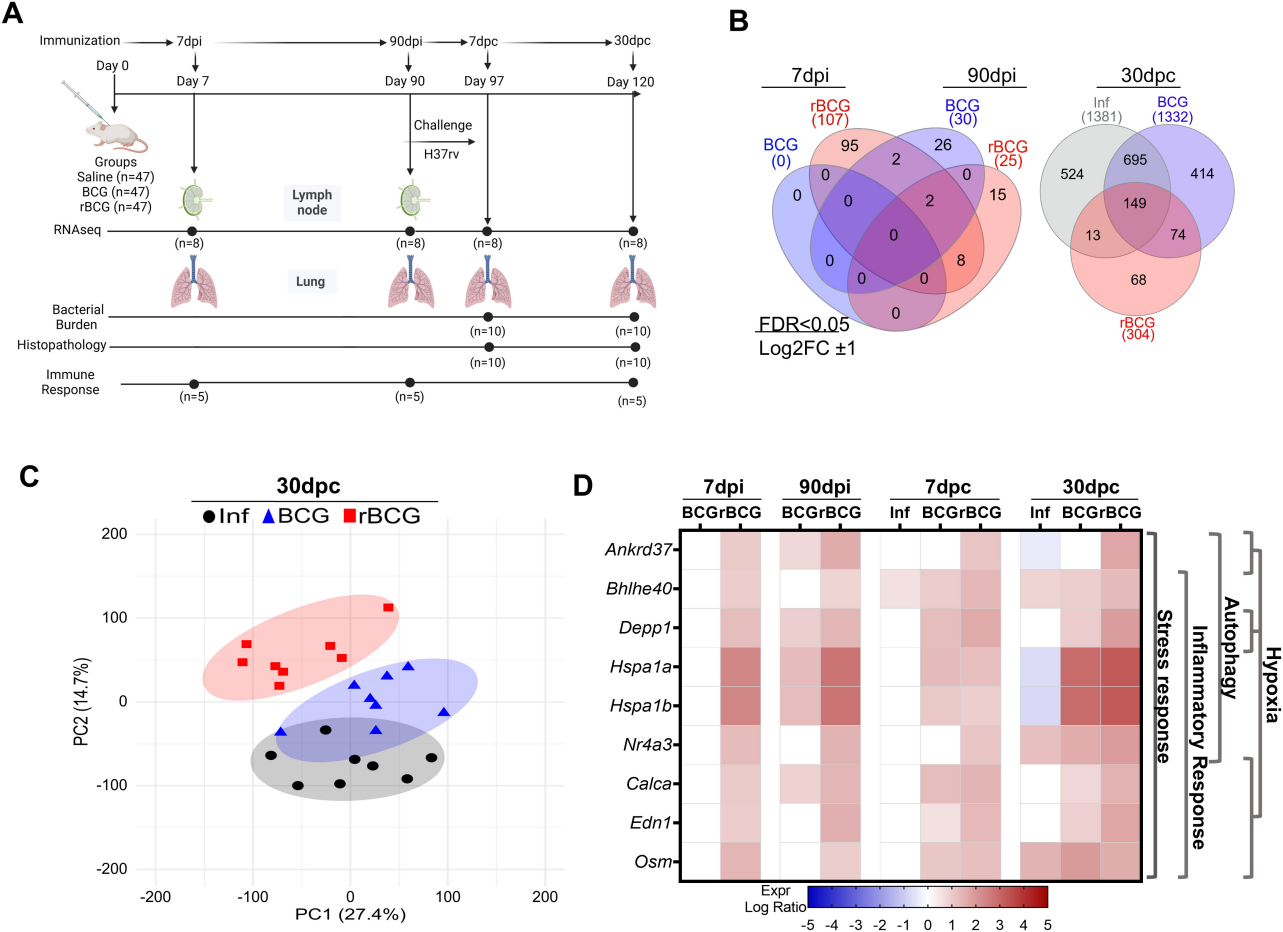
Immunization of mice with BCG or rBCG-LTAK63 induces distinct gene expression patterns. **(A)** Experimental design illustrating the distribution of groups, the time intervals, and the analysis performed. BALB/c mice were immunized with either BCG or rBCG-LTAK63 (rBCG); lymph nodes and lungs were collected at 7- and 90-dpi for RNA-seq transcriptome analysis. At 7- and 30-dpc, lungs were collected for RNA-seq, bacterial burden and histopathology assessments. Parallel groups of animals were used to evaluate immune responses at the same time points. **(B)** Venn diagrams depict the shared and unique genes expressed in the lungs of BCG or rBCG-immunized mice before (7 and-90-dpi) and after challenge (30-dpc). Differences were considered statistically significant with adjusted P < 0.05 and absolute log_2_FC >1 in DESeq2; only statistically significant data are presented. **(C)** Unsupervised principal components analysis of gene variation of all samples at 30-dpc. PCA displays components 1 (PC1) and 2 (PC2), indicating the percentage of variation in gene expression represented in each component. Dots represent individual lung samples: Infection only (Inf, n=8, black circles); BCG-immunized/challenged (BCG, n=8, blue triangles); rBCG-LTAK63-immunized/challenged (rBCG n=8, red squares) **(D)** Expression profile and associated functions of the nine genes detected as DEGs at all time points in rBCG-LTAK63-immunized/challenged animals in comparison with the BCG and Inf control groups. The color represents the log_2_ Fold Change (FC). Expression was considered significant when P < 0.05 and absolute log_2_FC >1). The side dendrogram presents the gene functions with the highest scores from the over-representation analysis.

Female 6-weeks old BALB/c mice (47/group) were immunized with rBCG-LTAK63 or BCG (1×10^6^ CFU/100 μL in PBS), or received saline, administered subcutaneously in their back, and challenged at 90 days. Animals were evaluated at 7- and 90-dpi and 7- and 30-dpc. Before challenge, lungs and draining lymph nodes were collected, while after challenge only lungs were evaluated. Lymph nodes were only used for RNA-seq. Throughout the experiments, the left lung and accessory lobe were designated for immune response analysis (n=5), transcriptomic assays (n=8), or independent RT-qPCR validation (n=6, see below). The right caudal lobe was allocated for histopathology and immunohistochemistry (n=5), while the right cranial and medial lobes were used for monitoring bacillary load (n=10). **Figure 1A** displays the general experimental design. The animals were euthanized via intraperitoneal injection of ketamine hydrochloride (300 mg/kg) and xylazine hydrochloride (30 mg/kg).

#### 2.3.1 Challenge and protection assays

All challenge experiments were performed at 90-dpi, through the intranasal route. Infection was carried out as described by Logan et al. (27). For the intranasal challenge, animals were anesthetized by intraperitoneal injection of ketamine hydrochloride (50 mg/kg) and xylazine hydrochloride (5 mg/kg). This dosage was chosen to ensure adequate sedation and tracheal relaxation, while minimizing respiratory depression. Further, *Mtb* H37Rv diluted in PBS at 1.25×10^4^ CFU/mL, from a previously prepared stock stored at -80°C. The different experimental groups were challenged intranasally with *Mtb* (500 CFU/40 µL). To confirm the bacterial load used, the lungs of a single mouse from each group were homogenized and plated on MB7H10 one day after inoculation.

Seven and thirty days after infection, the lungs of infected animals were collected to evaluate the immune response, pathology and bacillary load. To evaluate the bacillary load, the lung lobe was homogenized and plated on MB7H10. After 14–21 days of incubation at 37°C, the bacterial load was measured by quantifying the number of CFU.

#### 2.3.2 Cell isolation, stimulation and staining for cytometric analysis

To access the immune response at 7- and 90-dpi, and 30-dpc, lung lobes were perfused with PBS to recover blood cells, then digested with DNAse IV (30 g/mL) and collagenase II (0.7 mg/mL) at 37°C for 30min. The digested lungs were then meshed through 70 µm cell strainers to obtain single-cell suspensions. Erythrocytes were removed by incubating the cell suspension for 15min with RBC lysing solution (0.15 M NH_4_Cl and 10 mM KHCO_3_). The cell viability was determined using a Neubauer chamber with 0.2% Trypan Blue, the obtained viable lung parenchymal cells were adjusted to 1×10^6^ cells/mL, and 100 µL were plated in each well (1×10^5^ cells/well). Reagents were obtained from Sigma-Aldrich^®^, Merck KGaA, St. Louis, MO, USA. Cells were seeded in 96-well CellWells™ plates (Corning, Falcon, Durham, NC, USA). Samples used in the lymphocyte panel were stimulated with anti-CD3 and anti-CD28 (BD Pharmingen™, Franklin Lakes, NJ, USA), and 10 µg/well of BCG CFP (culture filtrate proteins), or 10 µg/well ConA (T cell responsive and proliferative control). Cells were incubated at 37°C and 5% CO_2_ for 4h, followed by the addition of Stop Golgi-Monensin solution (3 M; eBioscience, San Diego, CA, USA) and further incubation for another 10–12 h. Samples used in other panels were left unstimulated, followed by the addition of Stop Golgi-Monensin solution for 10–12 h.

Subsequently, cells were incubated with extracellular markers (described below) for 30min. Then, washed and fixed with Cytofix (BD Pharmingen™) for 30min. Cells were then permeabilized using the Mouse Cytofix/Cytoperm (BD Pharmingen™) by a 30-min incubation.

Antibodies used includes: anti-IFN-γ-FITC (clone XMG1.2, BD Pharmingen™), anti-CD4-PerCP (clone RM4-5, BD Pharmingen™), anti-IL-17-BV421 (clone 2F1, BD Pharmingen™), CD25-PE antibody (clone PC619, BD Pharmingen™), anti-FoxP3-AlexaFluor647 antibody (clone MF23A, BD Pharmingen™), anti-CD45 PerCP (clone 30-F11, Biolegend™, San Diego, CA, USA), anti-CD11c (APC clone N418, Biolegend™), anti-CD3 APC-Cy7 (clone 145-2C11, BD Pharmingen™), anti-CD11b BV421 (clone M1/70, Biolegend™), anti-F4/80 BV605 (clone BM8, Biolegend™), anti-Ly6G BV786 (clone 1A8, Biolegend™), anti-MHC-II FITC (clone M5/114.15.2, Biolegend™), anti-NK1.1 PE-CF594 (clone PK136, BD Pharmingen™), anti-Ly6C PE-Cy7 (cloneHK1.4, Biolegend™).

A BD FACS Canto II flow cytometer (BD Bioscience, Franklin Lakes, NJ, USA) was employed to assess a total of 100000 lung cells per sample, and the data were analyzed using FlowJoTM v10 Software. Gating positions for intracellular markers were defined using the full-minus-one (FMO) control strategy. Representative FACS plots illustrating the gating strategy for IFN-γ^+^/IL-17^+^ T cells, and the differences of monocytes, and neutrophils across groups are reported in **Supplementary Figures 8a–c**, respectively. A pan-panel for evaluating the frequency of immune cells from the innate and adaptive immune response was analyzed using t-distributed stochastic neighbor embedding (t-SNE). Briefly, CD45^+^ cells from all samples were concatenated and analyzed using t-SNE (28, 29). The algorithm’s default settings generated a two-dimensional projection of leukocytes based on eight distinct parameters corresponding to the markers used. To cluster the populations within the t-SNE projection, we employed the FlowSOM algorithm, configured to distribute the events into 10 distinct clusters (21).

#### 2.3.3 Cytokine production

Lung cells were cultured and stimulated as described above (2.3.2.) and incubated for 24h. The cell supernatant was then collected and maintained at -80 °C until use. After thawing, samples were centrifuged to remove debris, and the supernatants were analyzed for the presence of IFN-γ, IL-17 and IL-10, using a Cytometric Beads Array (CBA) mouse inflammatory reagent from BD Biosciences, according to the manufacturer’s protocol. Data acquisition was performed using the FACS Canto II cytometer, and subsequent analysis was conducted using the FCAP Array program (BD Biosciences).

### 2.4 Histopathological analysis

Lung tissue samples, from 7 and 30-dpc were fixed in 10% neutral buffered formalin overnight, preserved in 70% ethanol, paraffin-embedded, sectioned into 5-6 µm slices, and stained with hematoxylin and eosin (H&E). Pulmonary inflammation severity was assessed on digital images of H&E-stained lung sections acquired at 40× magnification with a Nikon Eclipse Ti-S microscope equipped with a DS-Fi1c digital camera. Image analysis was performed with the ImageJ software from the National Institutes of Health (Bethesda, MD, USA). Quantitative assessment included measuring the inflammatory area and determining the functional lung area through morphometric analysis of intra-alveolar regions. For qualitative evaluation, five random images at 40× magnification per lung lobule (totaling 25 images per treatment) were analyzed. Image processing involved converting 8-bit images, applying thresholds, and calculating the percentage of the measured area. The Color Deconvolution 2 plugin was used to visualize and separate nuclei and cytoplasm for leukocyte enumeration using the Cell Counter plugin, facilitating the enumeration of segmented and mononuclear nuclei.

### 2.6 Indirect immunohistochemistry

The lung tissue was deparaffinized and hydrated, and antigen recovery was performed in moist heat under citrate radiation for 15min. Endogenous peroxidase blockade was achieved using Dako Dual Endogenous solution (Dako, Carpinteria, CA, USA) and nonspecific sites were blocked with Dako Protein Block Serum-Free (Dako). The lung tissue slides were then incubated overnight for 18h in a humid chamber at 6 °C, with primary antibodies anti-IL-10 (Clone JES5-16E3, BD Pharmingen™), anti-IFN-γ (Clone XMG1.2, BD Pharmingen™) and anti-Gr1 (Clone 1A8, BD Pharmingen™) and then the sections were washed in PBS-T and the samples were incubated with the secondary antibodies. The secondary antibodies used were anti-rat IgG HRP (ThermoFisher Scientific, Waltham, MA, USA), according to the recommendations of the manufacturer (1:1000). The samples were incubated for 2h and developed with 3,3’-diaminobenzidine substrates (DAB, Sigma-Aldrich). The slides were mounted and observed under light microscopy using the Zeiss microscope model Axio Imager.M2; the images were captured with a Zeiss digital camera model AxioCam MRc and Olympus Stream Software (Olympus Life Science, Center Valley, PA, USA).

### 2.7 Transcriptome analysis

Groups of mice were immunized with BCG or rBCG-LTAK63 (1×10^6^ CFU/100 mL PBS) administered subcutaneously in their back with saline used as control. At 7 and 90-dpi, the left lung and accessory lobe from 10 mice were collected and stored in the RNAlater™ Stabilization Solution (ThermoFisher Scientific). At the same time points (7 and 90-dpi), lymph nodes were also collected and stored in RNAlater. The remaining animals were challenged at 90-dpi by intranasal infection as described previously. At 7 and 30-dpc, the lungs of infected animals were collected and stored as described above for sequencing.

### 2.8 RNA-seq data processing and analysis

Total RNA was extracted and purified individually from the left lung and accessory lobe samples or from lymph node samples stored in RNAlater (samples from 8 mice per group per time point) using the “RNA Extraction Protocol for Animal Tissue” Document number SOP-SMM-J003, Version A0 (BGI Genomics, Shenzhen, China) according to the manufacturer’s instructions. Purified RNA integrity was estimated by capillary electrophoresis (Fragment Analyzer; Agilent Technologies, Santa Clara, CA, USA), and samples showed RIN scores 7.0. Non-stranded libraries were constructed using the BGI in-house Library Prep Kit, and sequencing was performed on a DNBSEQ-G400 at BGI Genomics. Raw RNA-Seq data were deposited in the SRA repository at NCBI under the Project Accession number GSE278523.

Raw paired-end RNA-Seq data were subjected to quality control using FastQC (Babraham Bioinformatics, Cambridge, UK) (30). Trimmomatic (31) v.0.36 was used to remove the adaptors and filter low quality bases at termini (quality below 25) (LEADING:3 TRAILING:3 SLIDINGWINDOW:4:20 MINLEN:36). The filtered reads were aligned to the *Mus musculus* genome assembly Version GRCm39 using Bowtie2 v.2.3.5.1 (32), with “-very-sensitive” preset, keeping other parameters at default. The mapped and aligned reads were quantified to obtain the gene-level counts using featureCounts from the package Subread v.2.12 (33) with “is PairedEnd” and “allow MultiOverlap” parameters set to true and the remaining at default settings. Raw counts were processed using the Bioconductors package DESeq2 (34) v.1.36.0 in R v.4.2.3 and normalized using the DESeq method to remove the library-specific artifacts. Variance stabilizing transformation was applied to obtain normalized log2 gene expression values. Further quality control was performed using visual inspections of PCA results, box plots, histograms, and density plots. Differentially expressed genes (DEGs) were calculated using the Wald test in DESeq2 (34).

To reduce any housing, environmental, aging and other confounding variables, RNA-seq data from saline-immunized non-infected animals, collected at 7 and 90-dpi were pulled together as one group and used as baseline (Sal group n=16). Genes with absolute log_2_ Fold-Changes >1 and False Discovery Rate (FDR)-corrected *P value* <0.05, corrected for multiple testing using the Benjamini–Hochberg method, were considered differentially expressed. All analysis using RNA-seq data are displayed as *P value*.

The set of differentially expressed genes was analyzed with the use of Ingenuity Pathway Analysis^®^ IPA-Qiagen (QIAGEN Inc., Germantown, MD, USA; https://digitalinsights.qiagen.com/IPA). The main analysis used in this study was the Regulator Effects algorithm, which integrates the findings from the Upstream Regulator Analysis tab and the Downstream Effects Analysis (specifically, the Diseases & Functions analysis). This algorithm establishes links between upstream regulators, dataset molecules, and downstream functions or diseases affected in the dataset. The process develops a hypothesis that provides an explanation for the effect of activating or inhibiting a regulator located further up in the pathway on the expression of molecules located further down in the pathway.

### 2.9 Analysis of gene co-expression modules and networks

Gene co-expression modules in mouse lungs were analyzed using the Co-Expression Modules Identification Tool (CEMiTool) Bioconductor package (35). Default parameters were maintained. Enrichment pathways were established using MSigDB Hallmark, Canonical Pathways, and GO Biological Process. The interaction was built using MouseNet v2, which used 8154 microarray samples chosen from a pool of 76002 tested samples from GEO. This resulted in enhanced network inference techniques, allowing for the coverage of 17714 genes (nearly 88% of the coding genome) with 788080 linkages (36).

The degree of module expression activity was assessed by CEMiTool for each sample class via Gene Set Enrichment Analysis (GSEA). Prior to applying gene variance and expression filters, CEMiTool normalizes all input genes using the z-score gene method. Subsequently, the mean values are computed for each sample group. Next, each group was individually submitted to a pre-ranked Gene Set Enrichment Analysis (GSEA) using the default parameters. In the visual representations, red and blue colors indicate increased and decreased module activity, respectively. The Normalized Enrichment Score (NES) values from GSEA are directly proportional to the color intensity. The size of the circle shows the statistical significance of the FDR-corrected *P value*, which indicates the estimated probability that a gene set containing a specific NES is a false positive discovery (*P value* ≤ 1), hence the closer the value is to 0, the lower the probability of a false positive. The -log_10_ *P value* is the conversion of *P value* into inverse log_10_ value, therefore if the value is above 1, it is probably a true positive.

### 2.10 Tuberculosis gene module analysis

Tuberculosis-specific pathways and processes were collected from Moreira-Teixeira et al. and Singhania et al. (7, 37). Subsequently, gene set enrichment analysis was performed on the lung data of infected animals at 7 and 30-dpc, using these annotations (7, 37). The fold-change of each gene set was determined by calculating the average of the fold-change values of its constituent genes. Only modules with an absolute value of NES greater than 1.5 and a *P-value*<0.05 were considered significant.

### 2.11 Molecular degree of perturbation

The molecular degree of perturbation (MDP) method described by Pankla et al. (38) was used to assess transcriptomic perturbation across all groups and time points using unsupervised techniques to calculate the MDP, the normalized count data were submitted to https://mdp.sysbio.tools/analysis.

### 2.12 Cell-type specific enrichment analysis

Cell-type Specific Enrichment Analysis (CSEA) uses the Human Cell Landscape, Human Cell Atlas, and Tabula Sapiens systematic collections of tissue-cell-type expression signatures, derived from more than 5.5 million cells from 111 tissues and 1,355 tissue cell types (TCs), belonging to 61 adult and fetal tissues across 12 human organ systems (11 human organ systems + sensory system). The CSEA results were filtered for top 20 most enriched cell type and then filtered once more for immune-related cell type (39). CSEA was performed using https://bioinfo.uth.edu/webcsea/.

### 2.13 Cyclic AMP measurement

Intracellular cAMP levels were quantified in both RAW246.7 cells (*in vitro*) and whole lung tissue cells (*in vivo*) using acid extraction coupled with a commercially available enzyme-linked immunosorbent assay (ELISA) kit (Cayman Chemical, Ann Arbor, MI, USA), following the manufacturer’s instructions. For the *in vitro* assay, RAW264.7 macrophage-like cells were cultured in RPMI-1640 medium supplemented with 10% of heat-inactivated fetal bovine serum (FBS; Sigma-Aldrich^®^) and an antibiotic-antimycotic solution (100 U/mL penicillin, 100 µg/mL streptomycin; Thermo Fisher Scientific). Cells were seeded at a density of 1×10^5^/well in a 96-well plate and stimulated with a multiplicity of infection (MOI) of 1 of either BCG or rBCG-LTAK63 or left unstimulated. After 48h, the supernatant was aspirated and replaced with 100 μL of 5% trichloroacetic acid (TCA) in water. Plates were centrifuged at 1500 × g for 10min, and 50 μL of the resulting supernatant were used for cAMP assessment by ELISA. For the *in vivo* experiments, 50 µg of lung tissue was frozen in liquid nitrogen immediately after collection. The tissue was then thawed and homogenized using the TissueLyser II System (Qiagen Inc., Hilden, Germany) in 10 volumes (10 mL of buffer for each mg of tissue) of 5% TCA in water. The homogenate was then centrifuged at 1500 × g for 10min, and the resulting supernatant was carefully transferred to a clean test tube for use in the cAMP ELISA assay.

### 2.14 Real-time reverse transcription-polymerase chain reaction

At the specified time intervals, lung tissue was obtained from immunized animals. The lung tissue of animals collected at 30-dpc was lysed using TissueLyser LT with lysis buffer from the RNAase Kit (Qiagen). The total RNA was extracted following manufacturer’s instructions. The mRNA was transcribed into complementary DNA (cDNA) using the ThermoScript™ RT-PCR System (Invitrogen, Carlsbad, CA, USA) for First-Strand cDNA Synthesis. To validate the expression of the *Per1* and *Ccr2* genes (previously identified by RNA-seq), we performed quantitative polymerase chain reaction (qPCR) on a total of 8 sequencing samples pulled into 4 pools (each containing 2 samples), and we included 2 additional independent samples (not sequenced). The Applied Biosystems 7300 Real-Time PCR device was used with PowerSybr Master Mix (Applied Biosystems, Waltham, MA, USA). The expression of the *Mus musculus Per1* and *Ccr2* genes was measured and standardized using the ΔΔCt method, with the levels of *Gapdh* as the reference gene. Primers used were (synthesized by Thermo Fisher Scientific):

GAPDH Fw – CATCACTGCCACCCAGAAGACTG;
GAPDH Rv – ATGCCAGTGAGCTTCCCGTTCAG;
Per1_Fw – GAAACCTCTGGCTGTTCCTACC;
Per1_Rv – AGGCTGAAGAGGCAGTGTAGGA;
Ccr2_Fw – GCTGTGTTTGCCTCTCTACCAG;
Ccr2_Rv – CAAGTAGAGGCAGGATCAGGCT.

### 2.15 Autophagy assays and western blotting to detect LC3B

RAW264.7 macrophage-like cells were cultured and stimulated as described in 4.11. After 6, 12, or 24h, cells were lysed with ice-cold RIPA buffer (Thermo Fisher Scientific) supplemented with Complete Protease Inhibitor Cocktail (Roche, Basel, Switzerland). Equal volumes were loaded onto a 15% SDS-polyacrylamide gel, transferred to a PVDF membrane (Thermo Fisher Scientific), and blocked for 1h at room temperature with blocking buffer, i.e., PBS containing 0.05% Tween 20 (Sigma-Aldrich^®^) and 5% bovine serum albumin (Sigma-Aldrich^®^). Membranes were then incubated for 16h at 4 °C with rabbit anti-mouse-LC3B (1:1,000, clone 2775S) or anti-β-actin (1:1,000, clone 8457S; Cell Signaling Technology Inc., Danvers, MA, USA). Secondary antibody anti-rabbit IgG H&L (HRP) (1:3,000, clone ab6721, Abcam, Cambridge, UK) was incubated for 1h at room temperature. Reaction was detected using ECL Prime (GE HealthCare, Chicago, IL, USA) and images acquired with the LAS4000 digital imaging system (GE Healthcare).

### 2.16 Indirect pharmacological inhibition of Per1

To investigate the role of Per proteins in the dynamics of host-pathogen interactions, we treated animals with PF670462, a compound that indirectly inhibits the nuclear translocation of Per proteins. Mice received intraperitoneal injections of PF670462 at a dose of 30 mg/kg every other day. Treatment started on day 84 post-immunization and continued for 15 days following the challenge with *Mycobacterium tuberculosis* H37Rv, which occurred on day 90 post-immunization.

Throughout the experiment, animals were monitored daily for signs of distress or adverse effects related to the treatment or infection. On day 120 post-immunization (30-dpc), mice were euthanized as per established protocols. Lungs were collected to evaluate the effects of PF670462 on local immune responses and protection. Lung tissues were processed for bacillary load quantification and flow cytometry, following previously described methodologies.

### 2.17 Statistical analysis

Differences in bacillary load, gene expression (counts per million, CPM), pathology scores and histological analysis were analyzed using a one-way ANOVA followed by Tukey’s multiple *post-hoc* comparison test. Differences were considered statistically significant when *P* ≤ 0.05. Heatmaps, box plot graphs, Spearman’s and Pearson’s correlation coefficient, and statistical analyses were generated with GraphPad Prism 9. Calendar heatmaps and bubble heatmaps were created with https://app.rawgraphs.io/, while the experimental design figures were created with http://biorender.com.

## 3 Results

### 3.1 rBCG-LTAK63 induces an early and distinct expression profile in lungs and lymph nodes associated with stress response and autophagy

BALB/c mice were immunized s.c. with either BCG or rBCG-LTAK63, or administered saline as control, and RNA-seq was performed on the lungs and lymph nodes at 7- and 90-days post-immunization (dpi), and on the lungs at 7- and 30-days post-challenge (dpc) with 500 CFU i.n. dose of infectious *Mtb*. Bacterial burden and histopathology were also assessed post-challenge, and an independent cohort was used to evaluate lung immune responses (**Figure 1A**). Venn diagrams showing differentially expressed genes (DEGs) in immunized animals revealed that as early as 7-dpi a set of genes with significantly altered expression was detected only in rBCG-LTAK63-immunized animals in both the lymph nodes and lungs (**Figure 1B**, **Supplementary Figure S1A**). At 90-dpi, the number of DEGs was comparable between BCG and rBCG-LTAK63 in both organs; however, only 2 genes were shared in the lung and none in lymph nodes (**Figure 1B**, **Supplementary Figure S1A**).

It is noteworthy that, at 7-dpi, gene expression signatures associated with cellular stress responses (such as *Atf3*, *Ccl3*, *Hspa1a*, *Hspa1b* and *Hsph1*) were upregulated in both the lungs and lymph nodes of rBCG-LTAK63-immunized mice (**Supplementary Table 1**). Interestingly, *Hspa1a* and *Hspa1b* were still over-expressed in both organs at 90-dpi.

Following the intranasal challenge with *Mtb* at 7- dpc, lungs of mice immunized with BCG exhibited a higher number of DEGs (n=1011) as compared to both the infected (Inf, n=191) and rBCG-LTAK63-immunized mice (n=423), with a considerable overlap with the rBCG-LTAK63 group (284 DEGs, *i.e.*, 67% of the rBCG-induced DEGs) (**Supplementary Figure 1A**, **Supplementary Table 1**). At 30-dpc, the number of DEGs in the lungs of infected (Inf) mice or those previously immunized with BCG increased (n=1381 and 1332, respectively), with 695 shared between them (**Figure 1B**). Conversely, rBCG-LTAK63-immunized mice exhibited lower DEGs (n=304), with 68 genes exclusively expressed in this group (**Figure 1B**).

Molecular degree of perturbation (MDP) quantified how much each group deviated from the saline control. Scores at 30-dpc confirmed that the rBCG-LTAK63 group exhibited a substantial decrease in perturbation in comparison to the BCG or Inf groups (**Supplementary Figure 1B**). Similarly, Principal Component Analysis (PCA) showed that rBCG-LTAK63 group completely differentiates from the Inf and BCG groups at 30-dpc, which still significantly overlapped (**Figure 1C**). After immunization, at 7- and 90-, and early after challenge, at 7-dpc, there was an extensive overlap in the lung transcriptome patterns between the control and immunized groups (**Supplementary Figure 1C**).

Interestingly, the nine DEGs upregulated at all time points in rBCG-LTAK63-immunized mice are associated with cellular stress, inflammatory responses, hypoxia and autophagy (**Figure 1D**, **Supplementary Table 1**). Most of these genes showed decreased or no expression in BCG and/or the Inf groups (**Figure 1D**).

### 3.2 Early responses to rBCG-LTAK63 involve activation of the cAMP pathway in innate immune cells and increased autophagy

**Figure 2:**
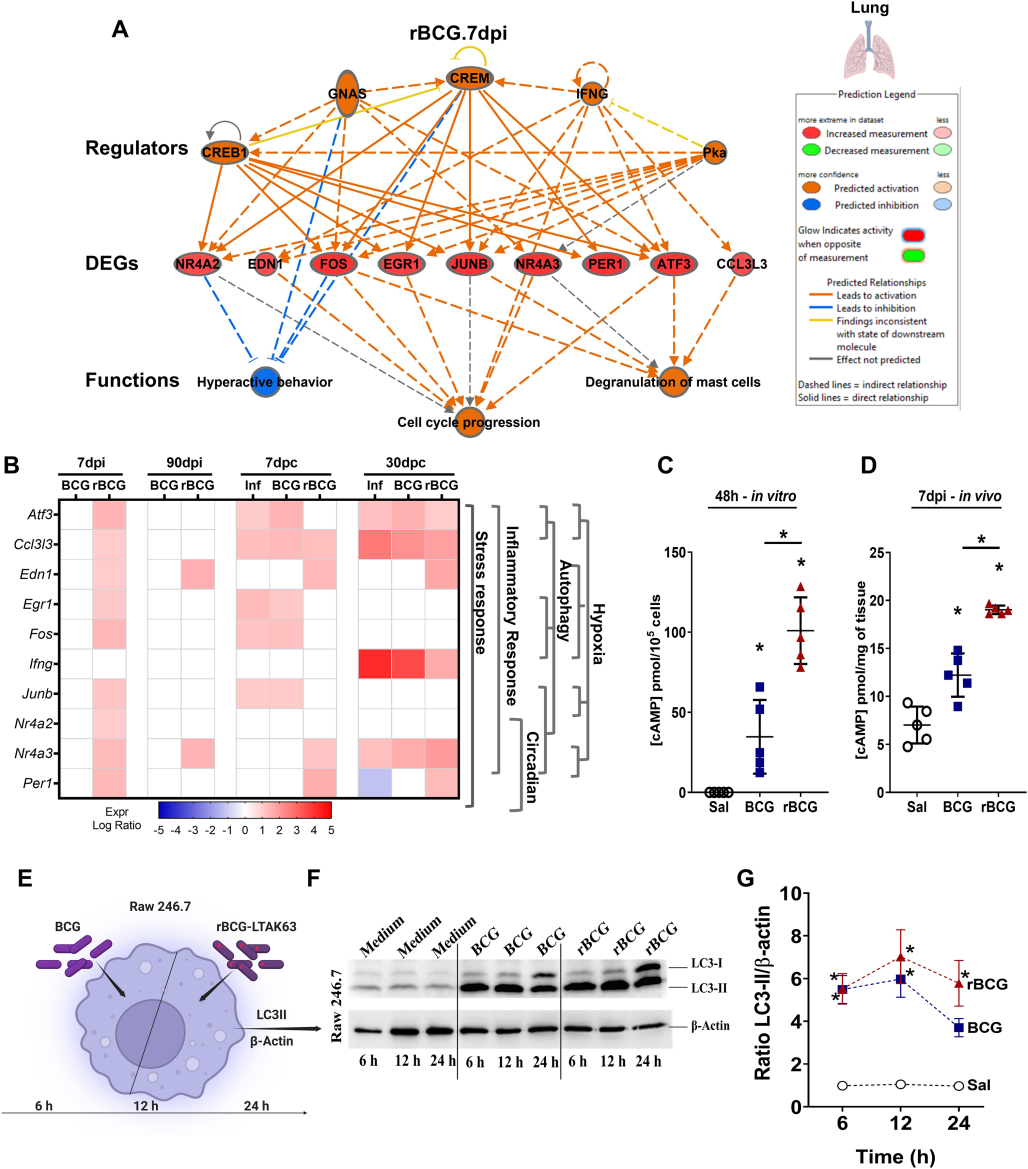
Early effect of rBCG-LTAK63 in the lungs of immunized animals suggests upregulation of cAMP-dependent genes and autophagy pathway. **(A)** IPA regulator effect diagram illustrating the correlation between DEGs at 7dpi, their predicted regulators and functions (*P value*<0.05, absolute Log_2_FC>1). Red symbols indicate upregulated genes, orange symbols are regulators or functions predicted to be upregulated, and blue symbols are predicted as downregulated. **(B)** Heatmap displaying the top genes identified in the regulator effects across different groups and time points (*P value*<0.05, absolute Log_2_FC > 1). The side dendrogram presents the gene functions with the highest scores from the over-representation analysis. **(C)** Quantification of cAMP in lung cells *in vitro* upon 48h of stimulation with BCG or rBCGLTAK63. **(D)** Quantification of cAMP levels in lung tissue from mice immunized with BCG or rBCG-LTAK63 at 7-dpi (n=5). **(E)** Experimental scheme of induction of autophagy in macrophages (RAW246.7 macrophage-like mouse cells) exposed *in vitro* to BCG or rBCG-LTAK63 (MOI 10:1). **(F)** RAW246.7 cells were collected at 6, 12, and 24h after stimulation, and evaluated for the conversion from LC3-I to LC3-II by Western blotting. **(G)** Saline (Sal), BCG and rBCG-LTAK63-induced autophagy in the RAW246.7 cells was evaluated as the ratio between LC3-II and β-actin, using densitometry analysis in ImageJ software (n=3/time point). Data shows mean values ± SD. An asterisk (*) above a group indicates a significant difference compared to the Inf (control) group (P < 0.05, one-way ANOVA). An asterisk with a horizontal bar denotes a significant difference between groups.

To understand the early mechanisms behind the enhanced protective properties of rBCG-LTAK63, we performed on Ingenuity Pathway Analysis^®^ (IPA-Qiagen) in the dataset at 7-dpi. IPA revealed that rBCG-LTAK63 immunization induced a single cluster of genes associated with the same “Regulator effects” in the lungs, including several genes involved in cell cycle progression and mast cell degranulation (**Figure 2A**). These same DEGs were also identified in an over-representation analysis performed with Enrichr, as involved in TNF-α signaling, reactive oxygen species (ROS) production and stress response (**Supplementary Figure 2A**). The Cell-type Specific Enrichment Analysis of genes (CSEA) indicated that these genes are mainly expressed in innate immune cells (**Supplementary Figure 2B**).

Multiple upstream regulators of these genes are induced by cAMP, such as the transcription factors PKA, CREB1, CREM1 and GNAS (**Figure 2A**). Gene expression analysis of the 7-dpi/IPA dataset across all time points confirmed an early response of genes involved in stress and inflammatory responses only in animals immunized with rBCG-LTAK63, many of which were reactivated after challenge (**Figure 2B**). Among these, again, several genes involved in autophagy were consistently upregulated: *Atf3, Erg1, Fos, Junb, Nr4a2 and Nr4a3*. Furthermore, genes involved in circadian rhythm regulation, *Per1* and *Nr4a3*, also showed early upregulation, followed by renewed induction post-challenge. Notably, *Per1* expression was downregulated in the Inf group at 30-dpc.

The levels of cAMP in the pulmonary tissue in response to the mycobacteria were examined in lung cells stimulated *in vitro* with BCG or rBCG-LTAK63. Increased cAMP production was observed, with rBCG-LTAK63 inducing significantly higher levels (**Figure 2C**). This elevation in cAMP levels was confirmed in the lungs of immunized animals at 7-dpi, once again with higher levels observed in the rBCG-LTAK63 group (**Figure 2D**).

Given that rBCG-LTAK63 elicited an increased cAMP response, inducing several genes related to autophagy (**Figures 1D**, **2B**), the functional induction of autophagy was investigated (40). Macrophage-like cells (the murine myeloid leukemia cell line RAW246.7) were stimulated with either BCG or rBCG-LTAK63, and cells were harvested at 6, 12, and 24h post-stimulation for evaluation by Western blot of LC3 induction and the conversion of LC3-I to LC3-II (both markers of autophagy). Elevated levels of LC3 (I and II) can be observed in response to both vaccines as compared to non-stimulated cells at 6 and 12h post-stimulation (**Figures 2E, F**). Notably, only rBCG-LTAK63-treated cells maintained a statistically significant increase in LC3-II as compared to non-stimulated cells at 24h (**Figure 2G**).

### 3.3 Co-expression analysis reveals early and persistent induction of IFN-γ response by rBCG-LTAK63

**Figure 3:**
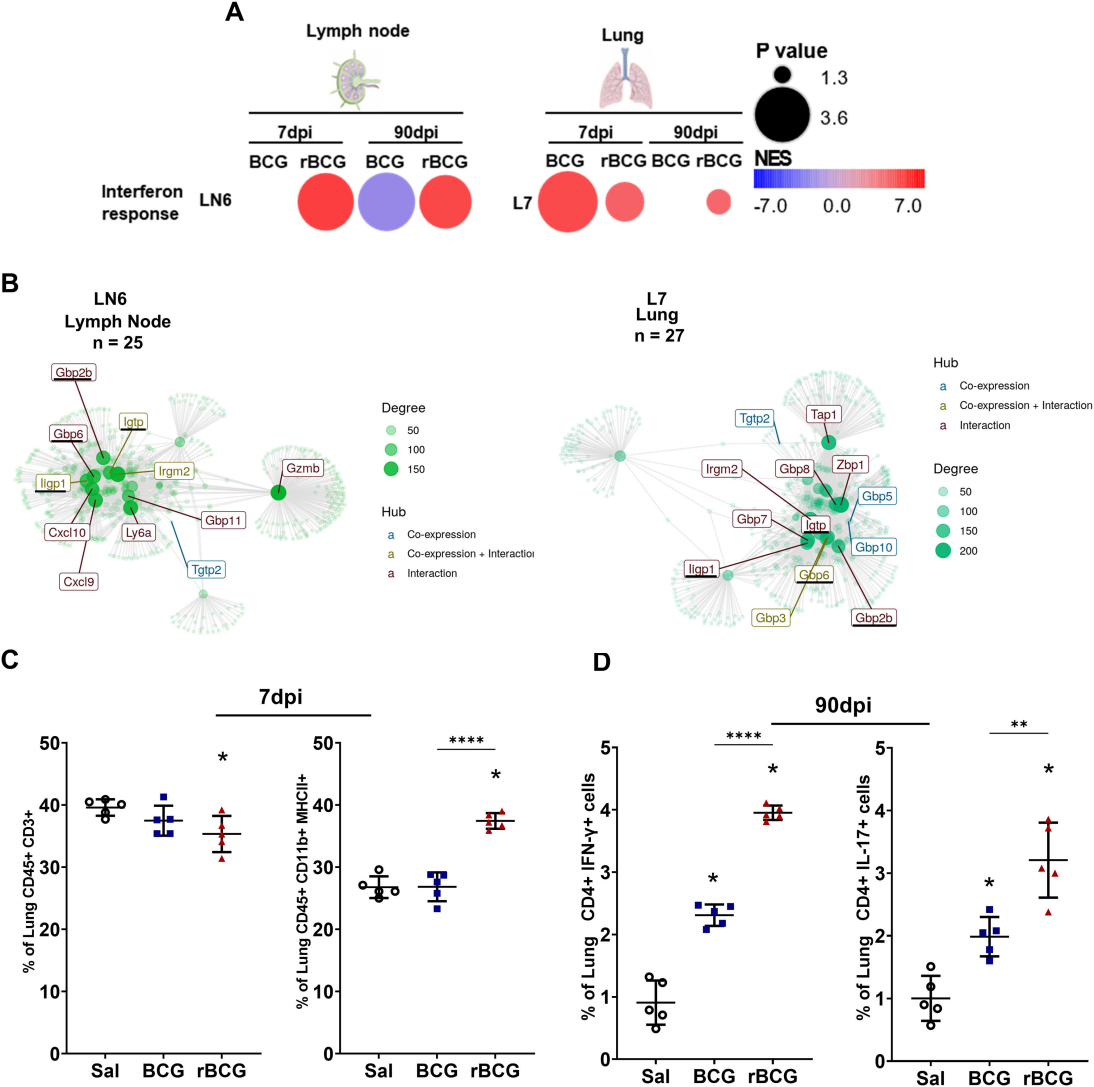
Co-expression analysis shows long-term regulation of IFN-γ-inducible genes by rBCG-LTAK63 immunization. **(A)** CEMiTool co-expression analysis was performed in BCG- and rBCG-LTAK63-immunized mice at 7- and 90-dpi. LN6: module 6 in lymph nodes; L7: module 7 in the lung. The color of the circles represents the Normalized Enrichment Score (NES), while the size indicates the -log_10_ *P value*. **(B)** The interaction maps show the connection between gene nodes in LN6 and L7, at 7 and 90-dpi, corresponding to the 25 and 27 genes of their respective modules, as well as additional genes linked by co-expression and/or interaction. Gene hubs’ origin are colored according to co-expression (colored blue), interaction (colored red) or both (colored green). The magnitude of the node is directly proportional to its degree of interactions. **(C)** At 7-dpi, the profile of immune cells in the lung was assessed to identify T cells (CD3^+^ CD45^+^) and bulk (CD45^+^ MHC-II^+^). **(D)** CD4^+^IFN-γ^+^ and CD4^+^ IL-17^+^ cells were assessed at 90-dpi in the lungs. Data shows mean values ± SD. An asterisk (*) above a group indicates a significant difference compared to the Inf (control) group (P < 0.05, one-way ANOVA); ** indicates P 0.01 and **** indicates P < 0.001. Asterisks with a horizontal bar denote a significant difference between groups.

We used CEMiTool analysis to i) detect modular patterns of gene co-expression (41), which were subsequently submitted to ii) gene set enrichment analysis (GSEA) and iii) protein-protein interaction (interactome) analysis, to identify potential hub genes. CEMiTool gene co-expression analysis performed on lymph nodes (LN) and lungs (L) at 7-dpi showed an enrichment and upregulation of IFN-related gene modules (LN6 and L7). In LN, the expression of module LN6 is induced and sustained in rBCG-LTAK63-immunized animals, while in the BCG group there is no change at 7-dpi, and it is downregulated at 90-dpi. In the lungs of rBCG-LTAK63-immunized mice, module L7 was enriched and upregulated at 7 and 90-dpi, while with BCG it was upregulated at 7-dpi but returned to baseline at 90-dpi (**Figure 3A**).

The interactome of genes enriched in the lymph nodes and lungs shared 11 co-expressed genes (**Supplementary Table 2**), with 4 hub genes identified (*Gbp2b*, *Gbp6*, *Ligp1*, and *Igtp*) (**Figure 3B**). These modules (LN6 and L7) contain genes enriched in pathways such as the response of type 2 interferon and formation of phagosomes (**Supplementary Table 2**). While several genes from LN6 and L7 are associated with IFN-γ (*Cxcl9, and Cxcl10)*, some of them can also be linked to Th17 (*Ptgs2)* responses (**Supplementary Table 2**). CSEA of these genes indicated that they are mainly expressed in neutrophils, NK cells and T cells (**Supplementary Figure 3D**).

Interestingly, the CEMiTool analysis also revealed that rBCG-LTAK63-immunized mice exhibited reduced enrichment of lung module 4 (L4) (involved in cell division, differentiation, and T cell activation) (**Supplementary Figures 3A, B**, **Supplementary Table 2**). Immunized mice exhibited a reduced frequency of lung T cells, alongside an increased proportion of CD45^+^MHC-II^+^ myeloid cells at this timepoint (**Figure 3C**). Despite the early T cell reduction, Th1 (CD4+ IFN-γ+) and Th17 (CD4+ IL-17+) cell frequencies increased in this group by 90-dpi (**Figure 3D**). This is consistent with the analysis of the immune landscape in the lungs at 7-dpi and at 30-dpc (**Supplementary Figures 4A, B**).

### 3.4 rBCG-LTAK63 induces rapid and persistent production of IFN-γ in both infiltrating cells and lung epithelial cells

**Figure 4:**
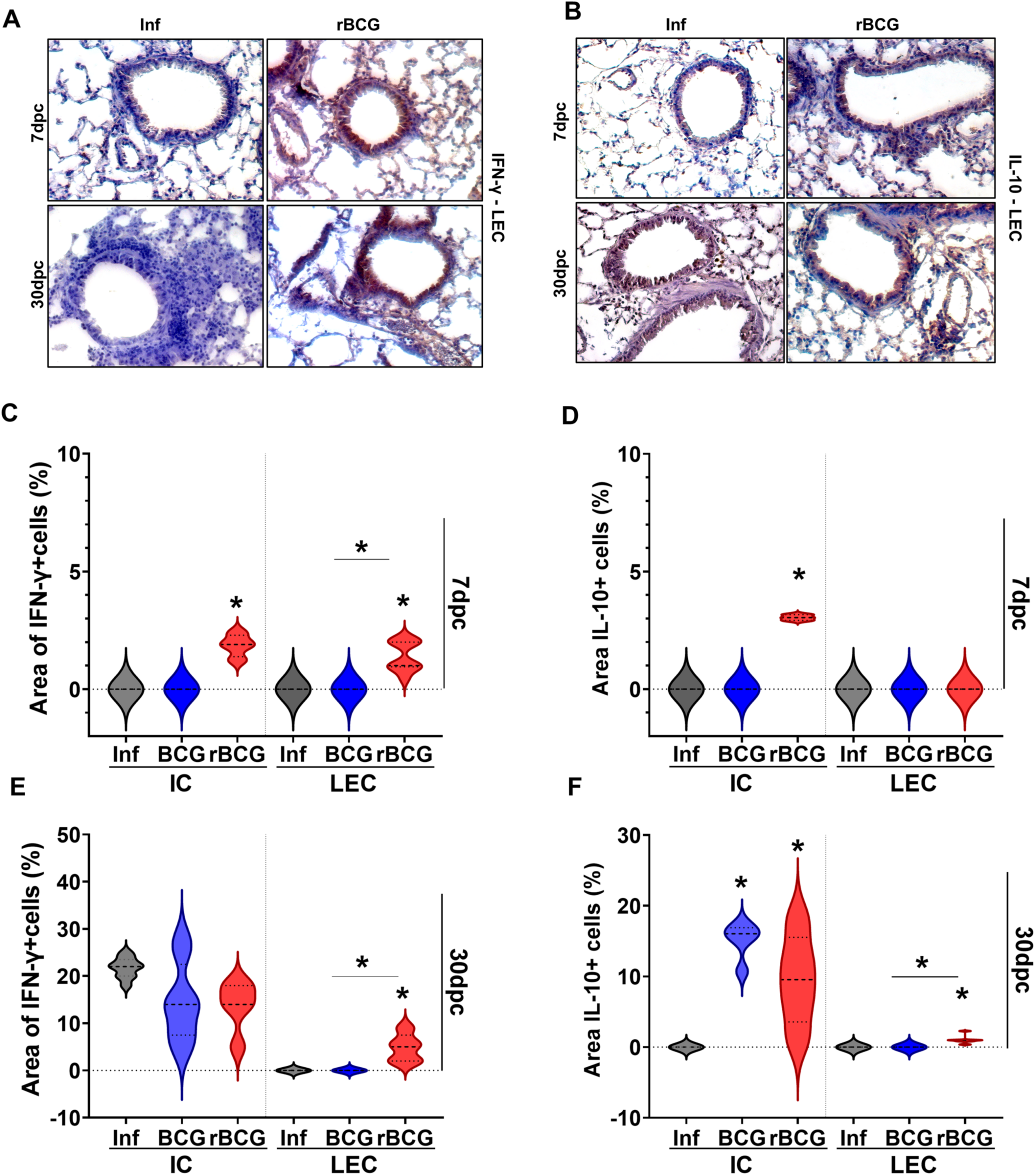
rBCG-LTAK63 induces increased IFN-γ+ cells in the infiltrating and epithelial cells in the lungs. Immunostaining of IFN-γ **(A)** and IL-10 **(B)** in lungs of immunized and control animals after Mtb challenge. The lung epithelial cells (LEC) (a,b) and infiltrated cells (IC) (**Supplementary Figure 6A**) were evaluated separately. The violin plots for IFN-γ **(C, E)** and IL-10 **(D, F)** display the percentage of positive area in comparison with total area of tissue (N=5), at 7 dpi and 30 dpi respectively. Data shows median values (dashed lines) and quartiles (dotted lines). An asterisk (*) above a group indicates a significant difference compared to the Inf (control) group (P < 0.05, one-way ANOVA). An asterisk with a horizontal bar denotes a significant difference between groups.

Given the sustained activation of IFN-associated gene modules in the lungs of rBCG-LTAK63-immunized animals, we investigated whether lung cells were more prone to produce IFN-γ immediately after infection. Immunostaining of lung tissue revealed enhanced production of IFN-γ (**Figure 4A**) and IL-10 (**Figure 4B**) already at 7-dpc and up to 30-dpc in the lungs of rBCG-LTAK63-immunized animals. At 7-dpc, IFN-γ+ cells were enhanced in both infiltrating cells (IC, **Supplementary Figure 6A**) and lung epithelial cells (LEC) (**Figure 4C**), while IL-10 was enhanced only in the IC of rBCG-LTAK63/Inf animals (**Figure 4D**). In contrast, BCG immunization had little impact on IFN-γ and IL-10 production at 7-dpc compared to the control group, in either IC or LEC (**Figures 4D, E**). At 30-dpc, IFN-γ production was increased in the IC of all groups and IL-10 in the immunized groups, but only rBCG-LTAK63/Inf animals showed increased IFN-γ+ and IL-10+ in LEC (**Figures 4A–D**).

### 3.5 rBCG-LTAK63 downregulates pathology-associated gene modules while maintaining protection-associated modules, leading to early and increased protection with lower inflammation.

**Figure 5:**
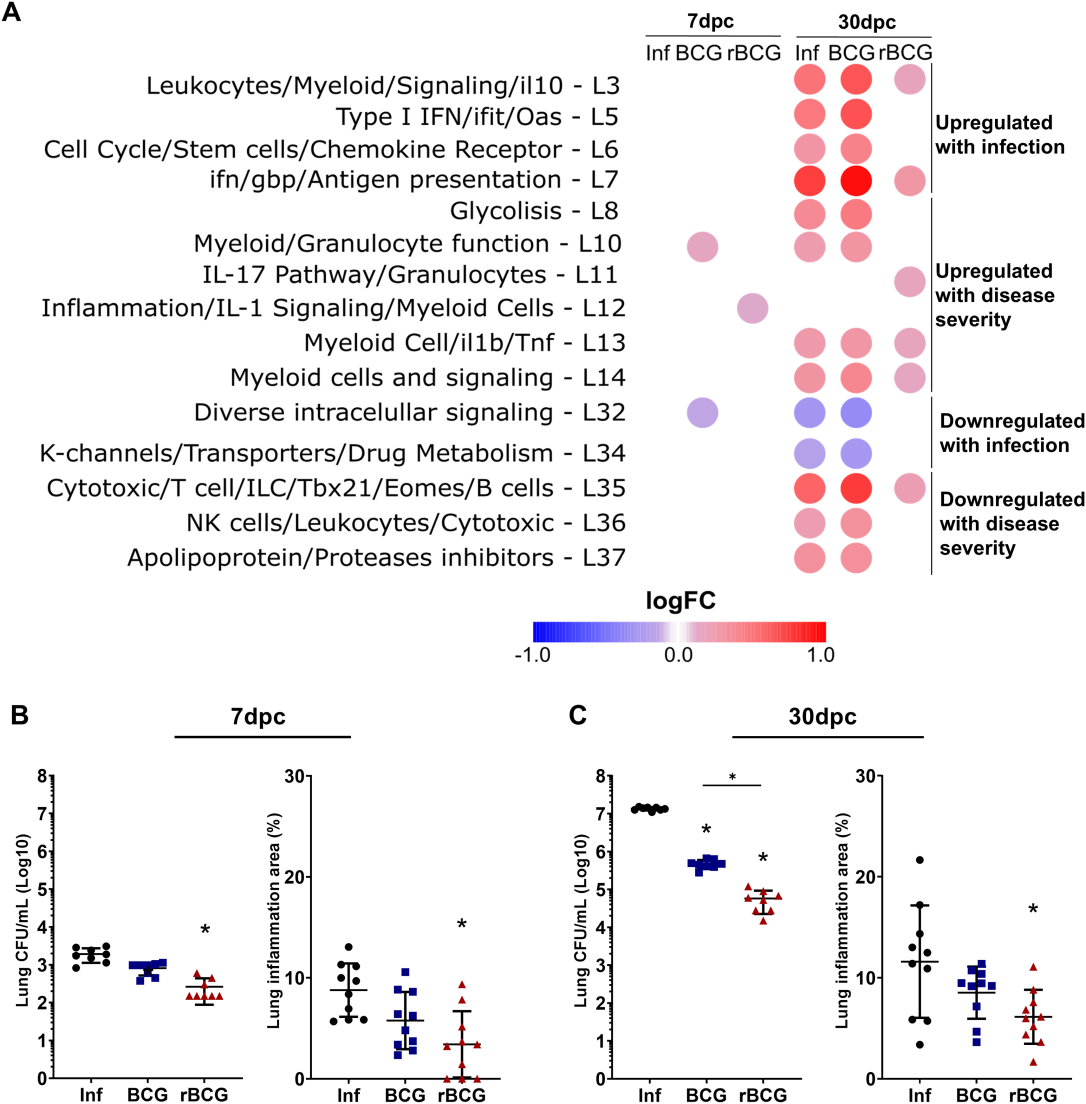
Effect of rBCG-LTAK63 on gene modules associated with pathology/severity of disease. **(A)** Gene module enrichment analysis was performed on our dataset, to assess the impact of BCG or rBCG-LTAK63 immunization on modules associated with disease severity (susceptibility, infection dose, or strain virulence) (39, 40). Titles on the left side describe the functions associated with the modules. Colors correspond to the average fold change relative to the Sal group. Only modules with well-defined functions and showing statistically significant differences (P > 0.05) are displayed. Assessment of CFU and functional area in the lungs of mice, after intranasal challenge with *Mtb*, at **(B)** 7 and **(C)** 30 dpi (n= 10). Data shows mean values + SD. Differences were considered statistically significant when P < 0.05 in a one-way ANOVA test). An asterisk (*) above the group indicates statistical significance compared to Sal (control), while an asterisk with a bar indicates differences between groups.

Previous studies have identified modular gene signatures associated with infection, pathology, strain virulence, and host susceptibility against tuberculosis across mice and human (7, 37). Thus, to investigate how BCG and rBCG-LTAK63 immunization modulates these responses, we compared our lung transcriptomic datasets using the annotated modular framework, categorizing modules as either upregulated or downregulated during infection, or as related to disease severity (**Figure 5A**; full analysis in **Supplementary Figure 6B**).

At 7-dpc, rBCG-LTAK63/Inf mice showed selective upregulation of the IL-1 signaling module (L12), with few other changes (**Figure 5A**). By 30-dpc, modules associated with infection and severity of disease (L3, L5, L6-L8, L10, L13, 14) were either unaltered or slightly upregulated, whereas in the infected (Inf) and BCG/Inf groups these modules were strongly upregulated (**Figure 5A**). Notably, rBCG-LTAK63/Inf mice preserved signaling modules (L32, L34), typically downregulated during infection, and selectively upregulated the IL-17 module (L11), while maintaining lymphocyte activation and ILC-related modules (L35).

Given the maintenance of protection-associated modules and the downregulation of pathogen-associated modules in the lungs of rBCG-LTAK63–immunized animals, we investigated whether, upon challenge, this would lead to an earlier onset of protection. Analysis of bacterial burden and lung inflammation demonstrated that protection induced by rBCG-LTAK63 was already visible at 7-dpc, as evidenced by a one log reduction in *Mtb* CFU and decrease in lung inflammation (**Figure 5B**, **Supplementary Figures 5A, C**). At 30-dpc, the increased protection induced by rBCG-LTAK63 was even more pronounced, with an approximately two-log reduction in lung CFU (**Figure 5C**). Furthermore, rBCG-LTAK63-immunized mice exhibited a reduced lung inflammation after *Mtb* challenge (**Figure 5B**, **Supplementary Figures 5B, C**).

### 3.6 rBCG-LTAK63 modulates inflammatory responses and TB-associated pathology through circadian rhythm gene modulation

**Figure 6:**
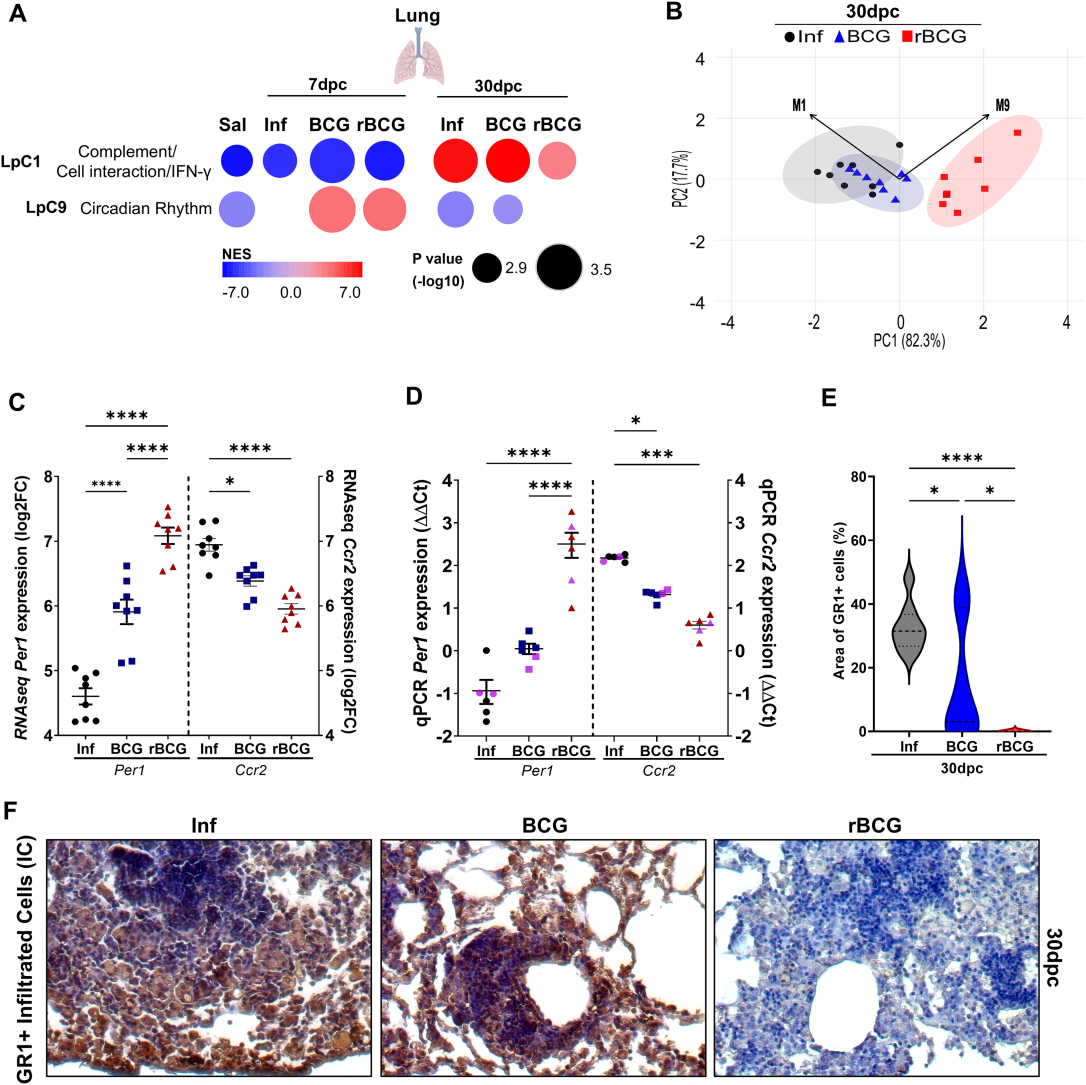
Post-challenge co-expression analysis suggests an association between rBCG-LTAK63 immunization and circadian rhythm: *Per1* gene expression shows high correlation with protection. Post-infection co-expression analysis using CEMiTool was conducted on normalized expression values from mice that were either non-immunized (Inf) or immunized with BCG or with rBCG-LTAK63 (rBCG) and then infected with *Mtb*. **(A)** Co-expression modules enrichment was analyzed at 7 and 30-dpc and compared with non-infected controls (Sal). Patterns of expression of modules 1 (LpC1) and 9 (LpC9) are shown, derived from the MSigDB Hallmark/Canonical Pathways/GO Biological Process dataset. The color of the circles represents the Normalized Enrichment Score (NES), while the size indicates the -log_10_ P value. **(B)** Principal Component Analysis (PCA) of the eigengene expression profiles of modules LpC1 (M1) and LpC9 (M9). Vectors represent the direction and relative strength (loadings) with which each module contributes to the principal components. The magnitude of each vector reflects the influence of the corresponding eigengene on the component’s structure. PC1 and PC2 accounted for 82.3% and 17.7% of the total variance, respectively. **(C)** The expression of *Per1* and *Ccr2* levels at 30-dpc were evaluated in the RNAseq dataset and validated by **(D)** qPCR using eight sequenced samples from RNA-seq assay combined in four pools (black circles, Inf; blue triangles, BCG; red squares, rBCG-LTAK63), and two additional independent samples (pink - not sequenced). qPCR was performed using specific mouse *Per1* and *Ccr2* primers (n=6). ΔΔCt relative to housekeeping *Gapdh* gene. **(E)** Immunostaining of Gr1^+^ cells in lungs of immunized and control animals at 30-dpc. Representative micrograph of each group showing the infiltrated myeloid cells expressing the Gr1 marker. **(F)** Evaluation of GR1-positive cells in the lungs of infected mice at 30-dpc. The violin plot displays the percentage of positive GR1+ staining relative to tissue area (n=5). Data show median values and quartiles. An asterisk (*) above a group indicates a significant difference compared to the Inf (control) group (P < 0.05, one-way ANOVA). (*** and **** indicate P < 0.05 and P < 0.001, respectively). Asterisks with a horizontal bar denote a significant difference between groups.

CEMiTool analysis at 30-dpc revealed that all groups showed enrichment and upregulation in module 1 (LpC1), although this was consistently less pronounced in the rBCG-LTAK63/Inf group; no significant differences were observed at 7-dpc (**Figure 6A**). Module LpC1 comprises 729 genes enriched in pathways associated with TB protection, but also pathology and disease progression, including IFN types 1 and 2, B and T cell activation and tolerance (**Supplementary Table 2**). Gene hubs within LpC1 (**Supplementary Figure 7A**) highlight the complex relation between protection and disease progression, exemplified by genes such as *Slc7a11* and *Cd274* (encoding PD-L1), which increase host susceptibility (42, 43), while *Tap1*, *Egr3* and *Cybb*, are associated with early recognition, antigen presentation and oxidative burst, thus contributing to host resistance (44–46).

At 7-dpc, expression of module 9 (LpC9), which is associated with circadian rhythm, is enriched and upregulated in both the BCG- and the rBCG-LTAK63/Inf groups, but not in the Inf group (**Figure 6A**). Notably, at 30-dpc, enrichment of this module decreased across all groups, but in the Inf and BCG/Inf groups the module was downregulated, whereas in the rBCG-LTAK63/Inf group it was not enriched (**Figure 6A**). This module comprises 26 genes, including core genes for the circadian rhythm process, such as *Arntl*, *Npas2*, *Per2*, and *Per3* (**Supplementary Figure 7B**).

Principal Component Analysis (PCA) was performed using the expression profiles eigengene vectors of modules LpC1 and LpC9. Notably, the vectors of both modules point in the same direction along PC2, but in opposite directions along PC1, suggesting divergent contributions to the primary axis of variation (**Figure 6B**). Interestingly, despite differing in the number of constituent genes, the similar lengths of their vectors indicate that both modules contribute comparably to the overall structure of the data.

PER1 is an important transcription factor in the circadian rhythm process (encoded by genes in LpC9), which also regulates *Ccr2* expression, involved in myeloid cell recruitment (47); the *Per1* and *Ccr2* genes are also present in LpC1. RNAseq derived expression level of *Per1* in the Inf group was lower than that in the immunized groups at 30-dpc (**Figure 6C**). Conversely, expression of the *Per1* gene was significantly upregulated at 7-dpi, 7-dpc (**Supplementary Figure 7C**) and at 30-dpc (**Figure 6C**), in the lungs of rBCG-LTAK63-immunized animals, with a more pronounced difference at 30-dpc. This was confirmed with independent samples by qPCR (**Figure 6D**). Correlation analysis at 30-dpc revealed a strong negative correlation between *Per1* expression and *Ccr2* expression or CFU (r < -0.8) (**Figures 6C, D**, **Supplementary Figure 7D**). In agreement, immunohistochemistry analysis at 30-dpc showed that the Inf and BCG/Inf groups presented a large area of GR1 staining (indicating the influx of myeloid cells), while the rBCG-LTAK63/Inf group did not (**Figures 6E, F**).

**Figure 7:**
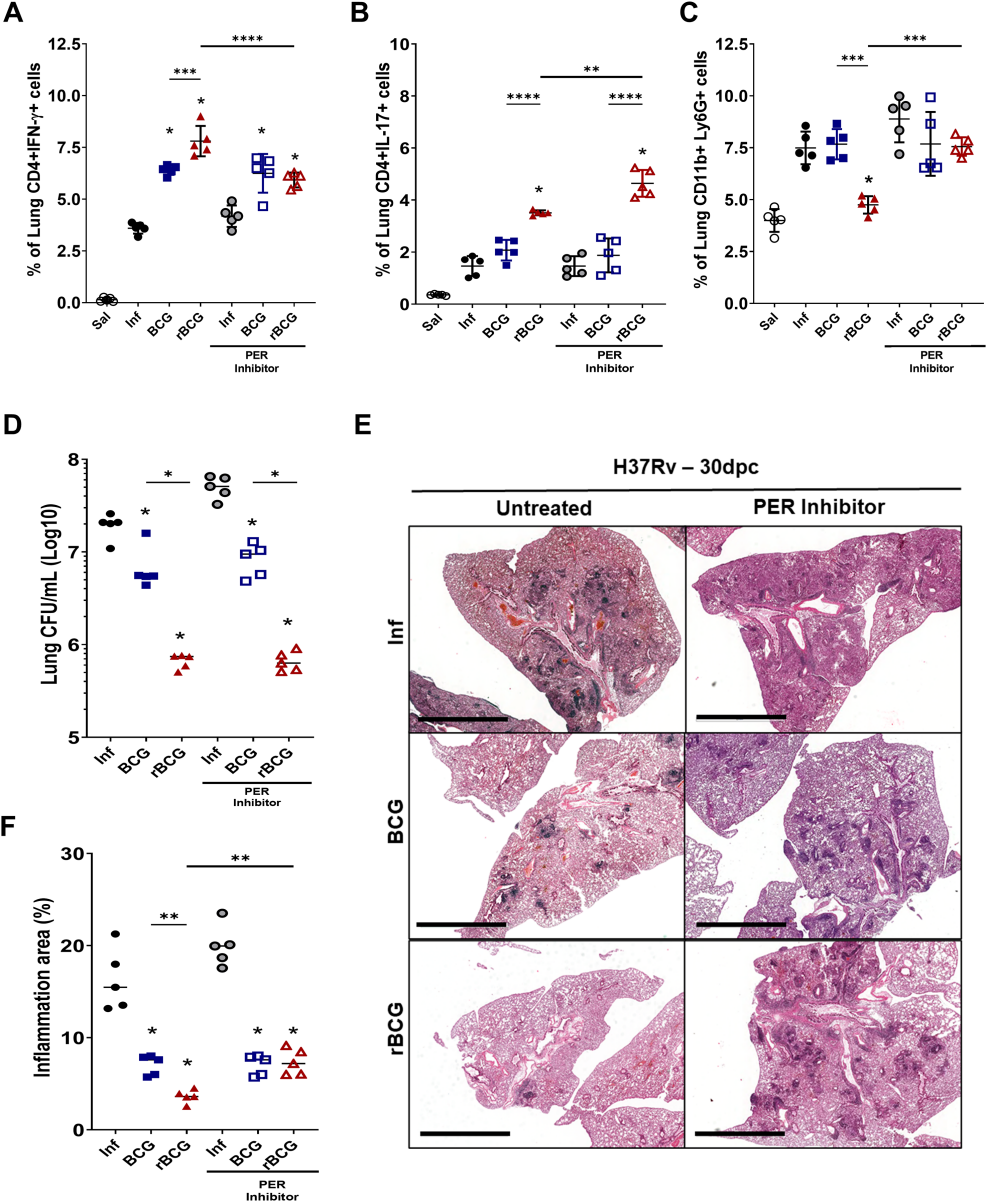
PER protein inhibition modifies Th1/Th17 responses induced by rBCG-LTAK63 without affecting protective efficacy, but reduces lung pathology. Immunized and control animals were either treated or not with the PER translocation inhibitor, PF670462, every other day from 7 days before to 14 days after *Mtb* challenge. At 90-dpi animals were challenged with 500 CFU of *M. tuberculosis* H37Rv. At 30-dpc, lungs were collected for analysis. The frequencies of **(A)** Th1 cells (CD4^+^IFN-γ^+^) and **(B)** Th17 cells (CD4^+^IL-17) and **(C)** neutrophils (CD11b+ Ly6C- Ly6G+) were assessed by flow cytometry. Data are presented as mean ± SD. An asterisk (*) above a group indicates a significant difference compared to the Inf (control) group (P < 0.05, one-way ANOVA); (**)(***)(****) indicate P < 0.01, P < 0.005 and P < 0.001, respectively. Asterisks with a horizontal bar denote a significant difference between groups. **(D)** Lung bacterial burden (CFU), and **(E)** evaluation of PF670462’s effect on *Mtb*-induced lung pathology in immunized mice. The cumulative pathology score reflects the sum of individual scores for bronchial, vascular, and interstitial inflammation. Data are presented as violin plots showing the mean and interquartile range. An asterisk (*) above a group indicates a significant difference compared to the Inf (control) group (P < 0.05, one-way ANOVA). An asterisk with a horizontal bar denotes a significant difference between groups. **(F)** Histopathological analysis of lung tissue (H&E staining) with representative micrographs from each group: infected (Inf), BCG-immunized, and rBCG-LTAK63-immunized mice, either untreated or treated with PF670462.

To investigate the role of Per1 and the circadian pathway in inflammation and protection, we used PF670462, which is an inhibitor of CK1δ/∈, a protein that facilitates PER proteins translocation to the nucleus (48). Thus, we indirectly decreased PER1 activity by using pharmacological inhibition of CK1δ/∈ with PF670462. As previously reported, rBCG-LTAK63 induces strong Th1 and Th17 responses (**Figures 7A, B**). Notably, pharmacological inhibition of PER proteins led to a reduction in the Th1 response (**Figures 7A**), while concurrently enhancing Th17 cell frequencies (**Figure 7B**), suggesting a potential role for PER1 in shaping the balance between these effector subsets. CCR2 is essential for recruiting myeloid cells, including neutrophils. Pharmacological inhibition of PER proteins also modulated neutrophil recruitment in rBCG-LTAK63-immunized mice (**Figure 7C**). rBCG-LTAK63 immunization reduced neutrophil recruitment, but treatment with PF-670462 showed increased neutrophil influx in the lungs. This suggests that PER1 is induced by rBCG-LTAK63 and may regulate neutrophil influx in infected lungs.

Finally, analysis of pulmonary bacterial burden indicated a modest increase (~0.3 Log_10_ CFU) in non-immunized/infected and PF670462-treated animals, implying that PER1 may contribute to innate resistance against *M. tuberculosis*. However, no significant changes in bacterial burden were observed in immunized animals treated with PF670462 (**Figure 7D**), suggesting that vaccine-induced protection is maintained despite circadian disruption. Interestingly, histopathological analysis of lung tissue showed that PER inhibition increased lung pathology in rBCG-LTAK63-immunized animals (**Figures 7E, F**), implicating circadian rhythm in regulation of pathology but not in the protective immune responses.

## 4 Discussion

The development of an improved TB vaccine is hindered by the lack of well-defined biomarkers of protection. Using a systems biology approach, we analyzed gene expression signatures to understand why expression of LTAK63 by BCG enhances immune responses and vaccine-induced protection in the mouse model. Immunization with rBCG-LTAK63 induced a unique set of DEGs in the lymph nodes and lungs as early as 7-dpi, which were not seen with BCG. The identified genes suggest a possible role of stress response (hypoxia) and autophagy in enhancing protective Th1/Th17 response. Unique DEGs were also present after *Mtb* challenge (7- and 30- dpc). Pathways associated with protection, such as T cells and Th1/Th17 adaptive responses, were enriched and persistently upregulated by the immunization with rBCG-LTAK63, while pathways associated with inflammation (7, 37) were less enriched in comparison to BCG or Inf groups. This modulation of gene expression by rBCG-LTAK63 after challenge correlated with increased immune responses, decreased bacterial burden and lower pathology-associated inflammation.

*Mtb* can delay T cell activation in the early phase of infection, which facilitates disease progression (49, 50). Noteworthy was the early and sustained gene signatures related to IFN-γ response in rBCG-LTAK63-immunized mice. Upon challenge, this sustained IFN-γ response drives the quick induction of IFN-producing cells in the lungs. Furthermore, lung epithelial cells were also found to produce IFN-γ, possibly being part of the pre-infection IFN-γ response signature. The IFN-γ responses are directly associated with protection. It was shown that even though phagocytes host *Mtb*, IFN-γ produced by non-hematopoietic cells, such as lung endothelium and epithelium are also source of this cytokine and actively participate in the protection against tuberculosis (51).

After *Mtb* challenge, the number of CD4^+^IFN-γ^+^ cells (Th1) in rBCG-LTAK63-immunized mice was still higher than in BCG-immunized animals, as well as gene modules associated with T cell and IFN-γ response. Likewise, previous results showed that mice immunized with rBCG-LTAK63 maintain increased numbers of CD4^+^ T cells expressing TNF-α, IFN-γ and IL-17 for longer periods (up to 180 dpi) (22). *Bhlhe40* gene, induced by rBCG-LTAK63, plays a critical role in downregulating IL-10 and upregulating IFN-γ in CD4^+^ T cells. Notably, *Bhlhe40* KO mice are more susceptible to *Mtb* infection (52–54). Upregulation of *Bhlhe40* may explain the enhanced Th1 immune response before *Mtb* infection and its sustained response afterwards.

Identifying genes that are differentially expressed at 7-dpi and regulators induced by rBCG-LTAK63 shed light on a possible role of cAMP, a pivotal second messenger in multiple pathways (55). Exposure of lung cells to rBCG-LTAK63 increases cAMP levels, as demonstrated by *in vitro* and *in vivo* experiments. Toxicity of LT is mainly through Gsα, a protein that upregulates adenylyl cyclase (ADCY1) and accumulates intracellular cAMP (56, 57). The mutation that generated the LTAK63 variant from the original and toxic LTA greatly reduced its ability to induce cAMP and its toxicity, in epithelial cells, macrophages, and dendritic cells *in vitro* (17, 18, 56, 58, 59). LTAK63’s residual activity may be the factor responsible for the increased cAMP levels induced by rBCG-LTAK63. Through activation of PKA, cAMP can regulate essential processes such as hypoxia adaptation, autophagy, and circadian rhythms (40, 60–62).

The interplay between PKA and HIF-1a has been previously shown, since the inhibition of PKA also inhibits HIF-1 (63). Heat-labile toxins have also been shown to induce HIF-1 (64), which can facilitate the upregulation of ADCY1 and PKA, also leading to elevation of cAMP levels (61, 65). rBCG-LTAK63-immunization induced the expression of genes associated with a hypoxic signature, such as *Depp1*, *Edn1*, *Bhlhe40*, and *Ankrd37*. *Depp1* (66, 67) and *Ankrd37* (68) are known to have a role in the initiation of hypoxia and oxidative stress-driven autophagy. Autophagy has been shown to play a role in protection against TB. Loss of autophagy function by lung macrophages results in acute susceptibility to a high-dose *Mtb* infection (69). Clinical *Mtb* isolates that can downregulate autophagy are associated with more severe TB disease (70). Likewise, the pharmacological induction of autophagy in mycobacteria-infected macrophages leads to an increased antigen presentation to T cells and a reduction in bacterial number (70). Therefore, stimulation of autophagy is associated with enhanced Th1 immune responses and protection against TB (69).

Additionally, the adjuvant properties of LT have been associated with inflammasome activation and release of IL-1β, as well as stimulation of APC, driving upregulation of activation markers (CD80, CD86 and MHC-II) (71). On one side, cAMP has been described to inhibit T cell proliferation (72), but on the other side, LT has also been described as a strong inducer of Th17 (73). Remarkably, our results reproduced all these effects in animals immunized with rBCG-LTAK63, with decreased T cell number and increased CD45^+^MHC-II^+^ cells in the lungs at 7-dpi, and induction of Th17 cells at 90-dpi.

Several pieces of evidence in the literature have suggested an interplay between circadian rhythm and TB: i) among the symptoms, cough is more frequent during daytime together with higher sputum bacillary load (74); ii) the metalloproteinase production by *Mtb*-infected cells has been shown to depend on *Bmal1*, the central gene in the circadian loop (75–77); and iii) it has been shown that BCG efficacy is influenced by the time of immunization (78). Circadian rhythm has also been shown to orchestrate the migration, proliferation, and differentiation of several immune cells (75, 76). Here we show that rBCG-LTAK63-activated circadian rhythm genes (*Per1* and *Bhlhe40*) and gene modules (LpC9), were negatively correlated with bacillary load and inflammation.

rBCG-LTAK63 immunization upregulated expression of *Per1*, related to circadian rhythm, possibly through production of cAMP (62). *Per1* showed a strong negative correlation with bacillary load, and its inhibition seems to impact CFU recovery in infected controls but not immunized mice. PER1 protein has also been associated with T cell polarization and inhibition of CCR2^+^ cell recruitment (47, 79). It has been shown that *Mtb* exploits the recruitment of CCR2^+^ cells, which are permissive host cells (80). Inhibition of CCR2^+^ cell recruitment may lead to lower susceptibility to *Mtb* infection (81) rBCG-LTAK63-immunized mice display reduced recruitment of Gr1^+^ cells (neutrophils, and other myeloid cells) in the lungs at 30-dpc. Accordingly, depletion of Gr1^+^ cells has been shown to decrease lung CFU upon challenge (82).

This study has some limitations. First, we challenged mice using the intranasal route. Appropriate models to evaluate protection are extremely important to establish protection biomarkers. The gold standard challenge model for TB is a low dose aerosol infection, as it mimics the natural infection and considers the fact that dose and route greatly impact TB pathogenesis (83). Nevertheless, the transcriptomic dataset induced by our intranasal model was remarkably similar to that previously reported for the aerosol challenge (7), supporting the reliability of the intranasal challenge to study active tuberculosis. As a second limitation, we have not performed experiments to determine the involvement of *Per1* using knock-out models to verify its influence in the cAMP levels, immune response, and protection assays. Another limitation of the study is in the generalization of vaccine-associated biomarkers. As the infection with *Mtb* does not generate protection against reinfection, the establishment of biomarkers is dependent on vaccines under development. As rBCG-LTAK63 is a unique vaccine, exploiting the adjuvant properties of the detoxified toxin, it probably exerts its protective effect through different mechanisms than other vaccines that also showed improved protection for TB, such as VPM1002 and MTBVAC. Finnaly, the rBCG-LTAK63 vaccine induces the expression of circadian cycle–related genes, which were associated with immune responses and vaccine-induced protection rather than with their classical role in the central nervous system. Although mice and humans display markedly different circadian behaviors and rhythms, the impact of these genes on the immune system may be comparable across species. Nevertheless, the translational potential of these findings should be carefully evaluated in future studies.

**Figure 8:**
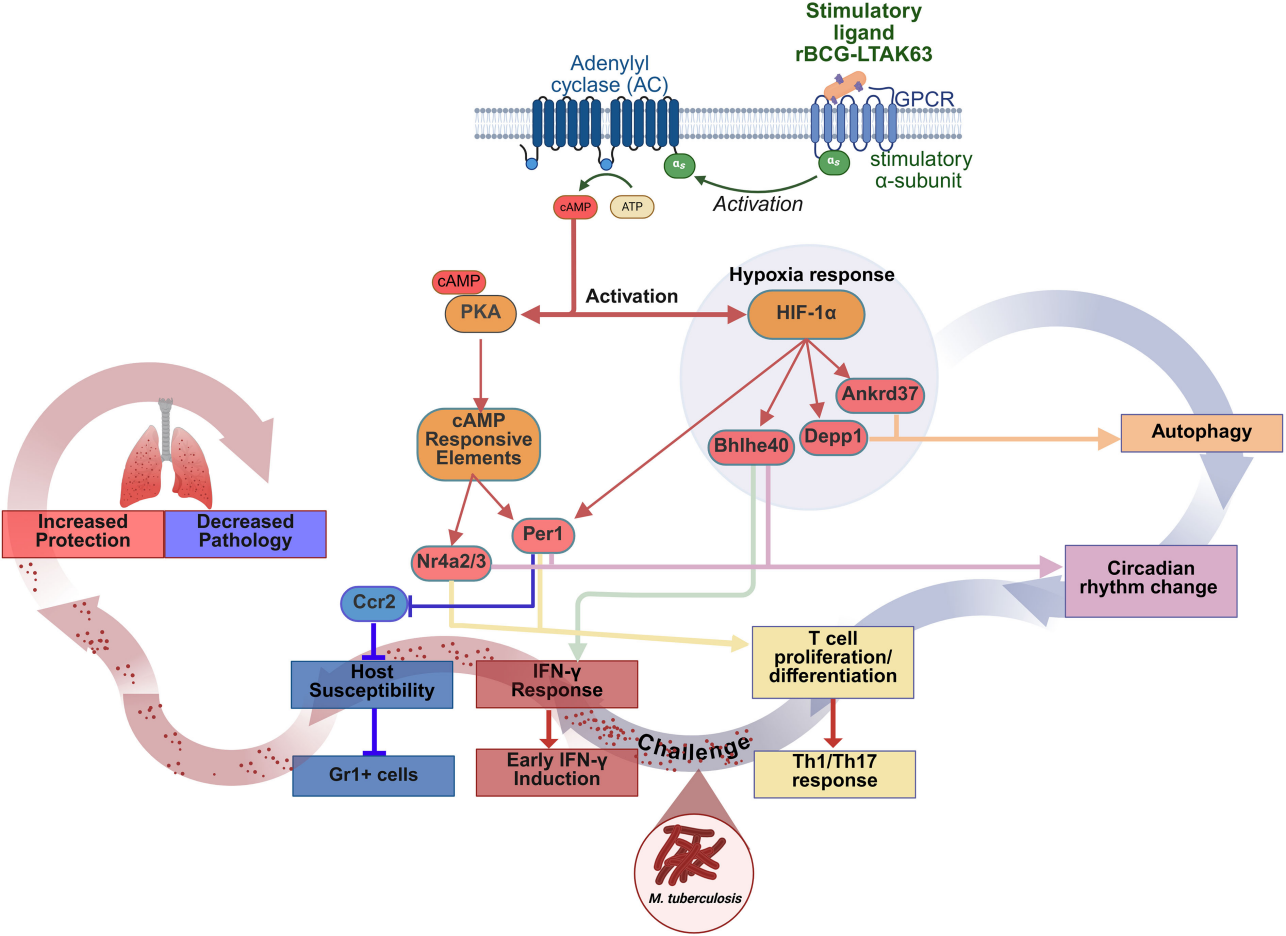
rBCG-LTAK63 orchestrates early protective responses mediated by cAMP/PKA/HIF-1a pathways inducing autophagy and enhancing IFN-γ responses and circadian rhythm regulation of pathology. Immunization with rBCG-LTK63 initiates a signaling cascade that involves the generation of cyclic AMP (cAMP), leading to the activation of protein kinase A (PKA). PKA activation results in the phosphorylation of cAMP-responsive element (CRE) transcription factors, such as CREB and CREM. Additionally, PKA and hypoxia-inducible factor 1 (HIF-1a) can reciprocally activate each other, which may influence circadian rhythm oscillations, thereby altering immune cell dynamics (*Nr4a2*/*3* and *Per1*). The interplay between circadian rhythm gene expression and hypoxia responses is also linked to autophagy (*Per1*, *Ankrd37*, *Depp1*), which can independently induce T cell differentiation into Th1 cells. Furthermore, hypoxia positively regulates the IFN-γ response through *Bhlhe40*. Collectively, these regulatory mechanisms orchestrate the inflammatory response and promote the development of a sustained IFN-γ response (increased memory), which, upon challenge, coordinates the immune response to eliminate bacteria and regulate the chemoattraction of Gr1^+^ cells. Ultimately, this process drives rapid bacterial clearance and mitigates pathology associated with disease progression.

On a whole, our hypothesis is that the residual cAMP-inducing activity of the genetically detoxified LTAK63 expressed in rBCG triggers PKA activation and upregulates the hypoxia response protein, HIF-1a (**Figure 8**). Activated HIF-1a promotes autophagy via induction of Ankrd37 and Depp1, while PKA activates cAMP-responsive elements, that upregulate Per1 and Nr4a2/3. These genes are implicated in both circadian rhythm- regulation and T cell proliferation/differentiation, enhancing Th1 and Th17 responses (**Figure 8**). Notably, PKA and HIF-1a activate each other, creating overlap between the autophagy and circadian rhythm pathways. Upon a challenge infection with *Mtb*, the IFN response is rapidly induced resulting in early production of IFN-γ. On the other hand, *Per1*, also induced upon challenge, inhibits recruitment of Gr1-positive/Ccr2-expressing myeloid cells; interestinlgly, mainly neutrophils were affected, thus reducing host susceptibility, and mostly controling the immunopathology caused by infection (**Figure 8**). Therefore the concerted action of autophagy induction and circadian regulation enhances protection while limiting lung pathology, acheived through preservation of protective immunity and reduction of detrimental inflammation.

Through the systems biology investigation of the rBCG-LTAK63-induced protection against TB in the mouse model, we provide data showing a role for cAMP-induction and autophagy promotion in the regulation of T cell responses linked to an early and persistent IFN response from immunization to challenge protection. Following challenge, the modulation through circadian rhythm genes results in the reduction of lung immunopathology, providing greater protection and safety.

## Data Availability

The primary data discussed in this study includes RNA-seq data, with supplementary tables containing raw data, lists of differentially expressed genes, gene expression values, and enrichment analysis results derived from CEMiTools. The sequencing files (RNA-Seq) have been deposited in the NCBI Sequence Read Archive (SRA) under Project Accession number GSE278523. Additional access to non-submitted data will be granted upon request, subject to the approval of a data access agreement. The data can be shared with researchers for further analysis, but it will be made available only to those who comply with the terms outlined in the data access agreement.

## Supplementary material

Supplementary Table 1 Comprehensive dataset of differentially expressed genes in lymph nodes (Excel Tab 1: 7- and 90-dpi) and lungs (Excel Tab 2: 7- and 90-dpi, 7- and 30-dpc). The table also provides the complete gene expression profiles for all samples in the lymph nodes (Excel Tab 3) and lungs (Excel Tab 4).

Supplementary Table 2 Results of the module over-representation analysis conducted using CEMiTool. Excel Tab 1 presents the pathways enriched for genes in modules L4, L6, and L7, derived from the dpi analysis of the lung tissue dataset. Excel Tab 2 contains the pathways enriched for genes in module LN6, from the dpi analysis of the lymph node tissue dataset. Excel Tab 3 displays the pathways enriched for genes in modules LpC1 and LpC9, based on the dpc analysis of the lung tissue dataset.
