# Supplementary material for: rBCG-LTAK63 enhances protection against tuberculosis by inducing autophagy and circadian gene regulation
Source: Front Immunol. 2025 Oct 24;16:1695560. doi: 10.3389/fimmu.2025.1695560 (PMC12592191; doi:10.3389/fimmu.2025.1695560)
Supplement: Supplementary file 3 [file DataSheet1.pdf]

Supplementary Figures

rBCG-LTAK63 Enhances Protection Against Tuberculosis by Inducing Autophagy and Circadian Gene Regulation

Lázaro M. Marques-Neto[a], Monalisa M. Trentini[a], Ana Carolina R. Moreno[a], Silas F. Eto[b], Ana Carolina O. Carvalho[a], Almiro P.S. Neto[c], Murilo S. Amaral[d], André G. C. Martins[e,f], André N. A. Gonçalves[f,g], Ana Marisa Chudzinski-Tavassi[b], Alex I. Kanno[a], Aldo Tagliabue[h], Diana Boraschi[h,i], Sergio Verjovski-Almeida[d,j], Helder Nakaya[e,g] Leonardo P. Farias[c], Pablo I.P. Ramos[k], Luciana C. C. Leite*[a].

[a] Instituto Butantan, Laboratório de Desenvolvimento de Vacinas. 05503-900, São Paulo, SP, Brazil.

[b] Instituto Butantan, Center of Excellence in New Target Discovery (CENTD).

[c] Instituto Gonçalo Moniz, Laboratório de Medicina e Saúde Pública de Precisão (MeSP2), Fundação Oswaldo Cruz (FIOCRUZ-BA). 40296-710, Salvador, BA Brazil.

[d] Instituto Butantan, Laboratório de Ciclo Celular, 05503-900, São Paulo, SP, Brazil.

[e] University of São Paulo. Department of Clinical and Toxicological Analyses, School of Pharmaceutical Sciences, 05508-220, São Paulo, SP, Brazil.

[f] Institute for Technological Research, Micromanufacturing Laboratory, São Paulo, Brazil. 05508-070

[g] Hospital Israelita Albert Einstein, 05652-900, São Paulo, SP, Brazil.

[h] Shenzhen Institutes of Advanced Technology (SIAT), 518055, Shenzhen-Guangdong, China.

[i] Shenzhen University of Advanced Technology (SUAT), 518055, Shenzhen-Guangdong, China.

[j] Universidade de São Paulo, Instituto de Química, Departamento de Bioquímica, 05508-000, São Paulo, SP, Brazil.

[k] Instituto Gonçalo Moniz, Centro de Integração de Dados e Conhecimentos para Saúde (CIDACS), Fundação Oswaldo Cruz (FIOCRUZ-BA). 40296-710, Salvador, BA, Brazil.

*Luciana Cezar de Cerqueira Leite

Email: luciana.leite@butantan.gov.br

Postal address: Laboratório de Desenvolvimento de Vacinas, Instituto Butantan, Avenida Doutor Vital Brasil, 1500, 05503-900, São Paulo, SP, Brazil.

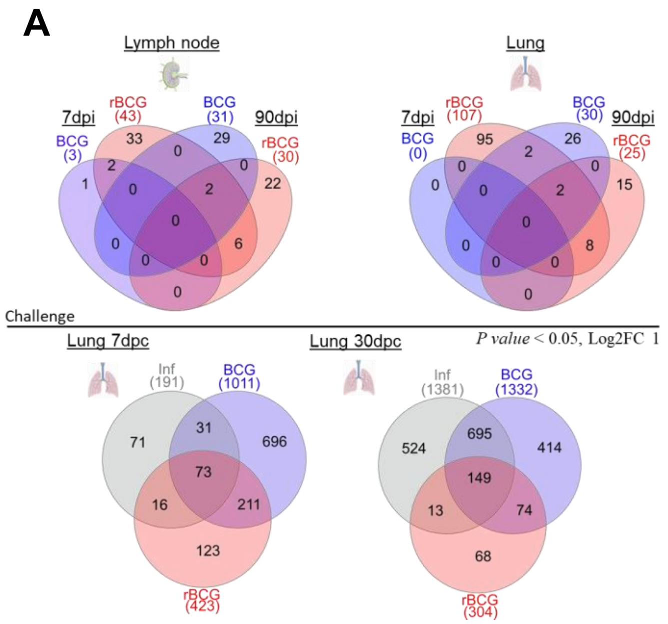

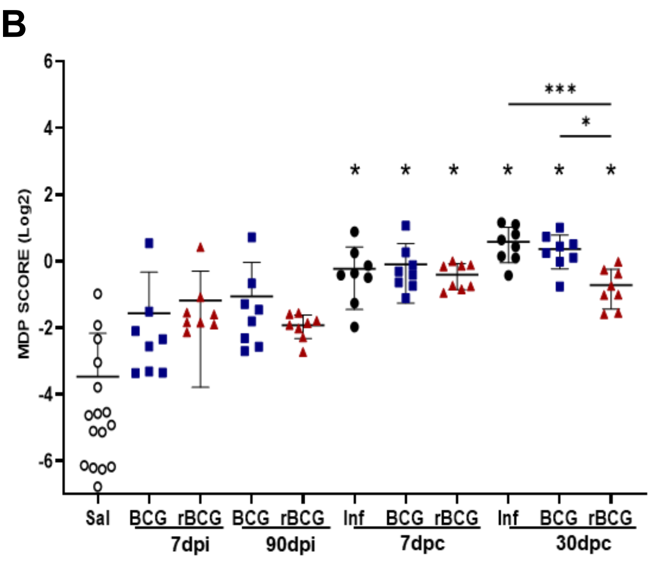

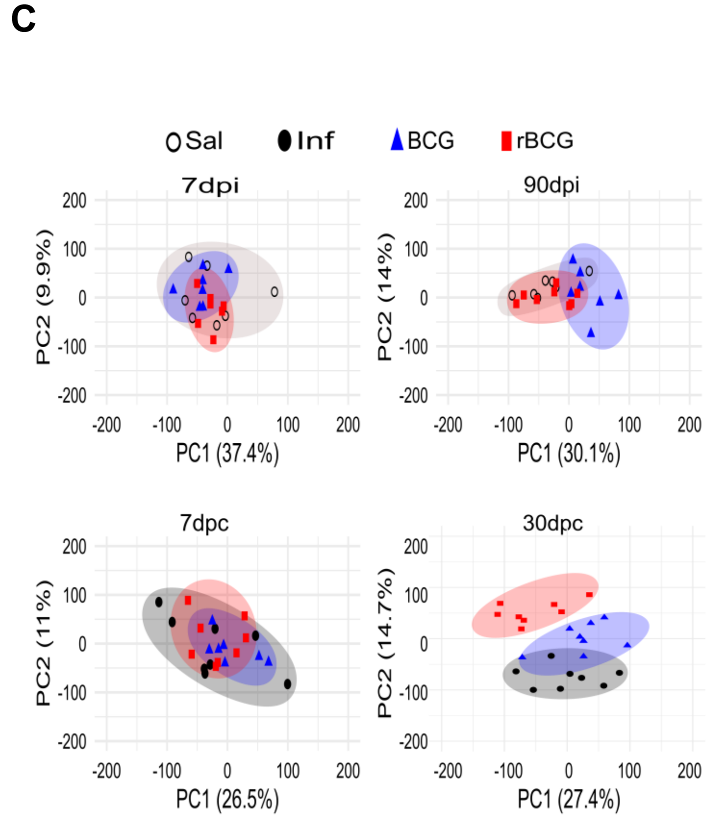

Supplementary Figure 1. Evaluation of gene expression in the lungs. (A) Venn diagrams depict the shared and unique genes expressed in the lymph nodes and lungs of BCG or rBCG-immunized mice before and after challenge (P < 0·05, absolute log2FC >1 **(B)** Evaluation of gene expression in the lungs by Molecular Degree of Perturbation (MDP). Unsupervised analysis was performed with all experimental groups and time points using normalized gene expression of all genes in the lungs by MDP (Sal group n=16, other groups n= 8). Data are presented as mean ± SD. Differences were considered statistically significant when P <0·05 in one-way ANOVA. An asterisk (*) above the group indicates statistical significance compared to Sal (control), while asterisks (*) or (***), P <0·05 or P>0·005, respectfully) with a bar indicates differences between other groups. **(C)** Principal component analysis displays components 1 (PC1) and 2 (PC2), indicating the percentage of variation in gene expression represented in each component. Symbols represent individual lung samples: Saline control (Sal, n=16), open circles, light gray shade; Infection only (Inf, n=8), black circles, dark gray shade; BCG-immunized (BCG, n=8), blue triangles, blue shade; rBCG-LTAK63-immunized (rBCG, n=8), red squares, red shade.

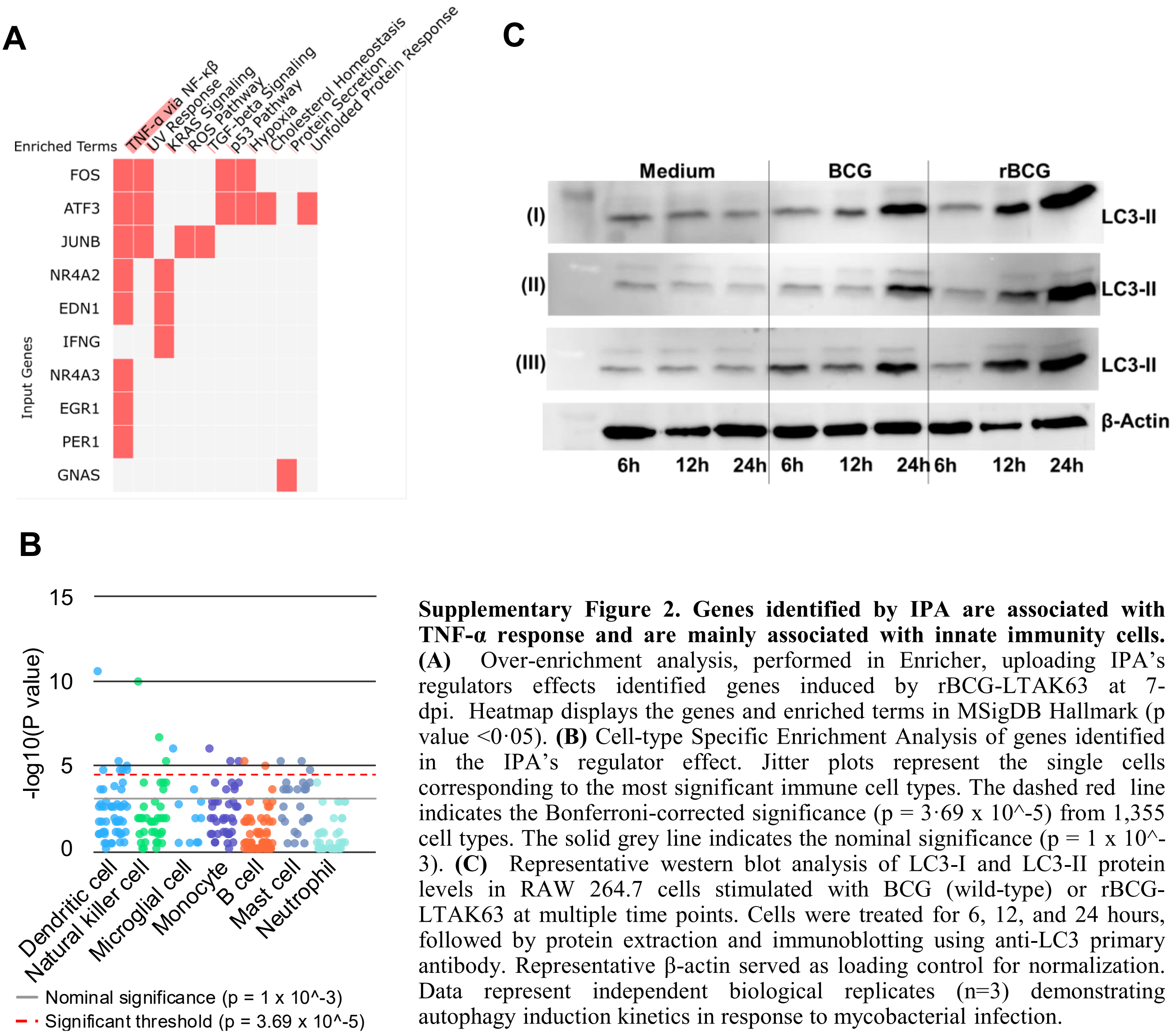

Supplementary Figure 2. Genes identified by IPA are associated with TNF-α response and are mainly associated with innate immunity cells. **(A)** Over-enrichment analysis, performed in Enricher, uploading IPA's regulators effects identified genes induced by rBCG-LTAK63 at 7-dpi. Heatmap displays the genes and enriched terms in MSigDB Hallmark (p value <0·05). **(B)** Cell-type Specific Enrichment Analysis of genes identified in the IPA's regulator effect. Jitter plots represent the single cells corresponding to the most significant immune cell types. The dashed red line indicates the Bonferroni-corrected significance (p = 3·69 x 10^-5) from 1,355 cell types. The solid grey line indicates the nominal significance (p = 1 x 10^-3). **(C)** Representative western blot analysis of LC3-I and LC3-II protein levels in RAW 264.7 cells stimulated with BCG (wild-type) or rBCG-LTAK63 at multiple time points. Cells were treated for 6, 12, and 24 hours, followed by protein extraction and immunoblotting using anti-LC3 primary antibody. Representative β-actin served as loading control for normalization. Data represent independent biological replicates (n=3) demonstrating autophagy induction kinetics in response to mycobacterial infection.

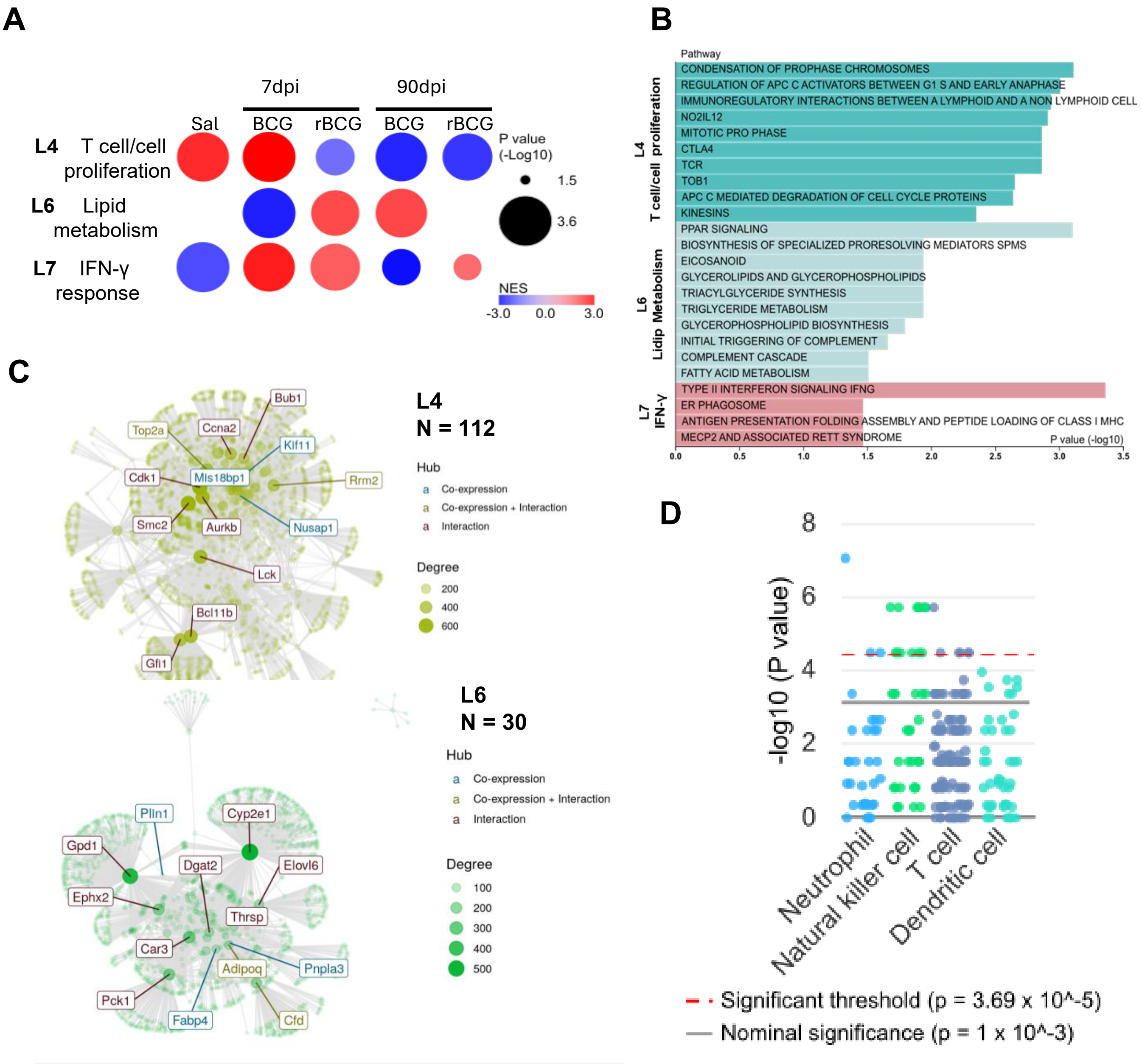

Supplementary Figure 3. Co-expression Analysis shows rBCG-LTAK63 regulates early cell proliferation and differentiation, and long-term IFN-γ inducible genes. Normalized expression values obtained from BCG or rBCG-LTAK63 immunized mice and the control Saline group at 7 and 90 dpi, were subjected to co-expression analysis using CEMiTool. **(A)** Different patterns of expression associated with immune response were observed in the displayed modules. The color of the circles represents the Normalized Enrichment Score (NES), while the size indicates the -log10 P value. **(B)** The bar plot represents the integration of over-representation analysis conducted on the modules derived from the Canonical Pathways, in which only pathways exhibiting significant enrichment (FDR < 0·05) are included. **(C)** Integrated network displaying interaction data for modules L4 and L6. The number of genes identified in each module (N) is indicated below the module title. **(D)** Cell-type Specific Enrichment Analysis of genes from modules LN6 and L7. Jitter plots represent the single cells corresponding to the most significant immune cell types. The dashed red line indicates the Bonferroni-corrected significance (p = 3.69 x 10^-5) from 1,355 cell types. The solid grey line indicates the nominal significance (p = 1 x 10^-3).

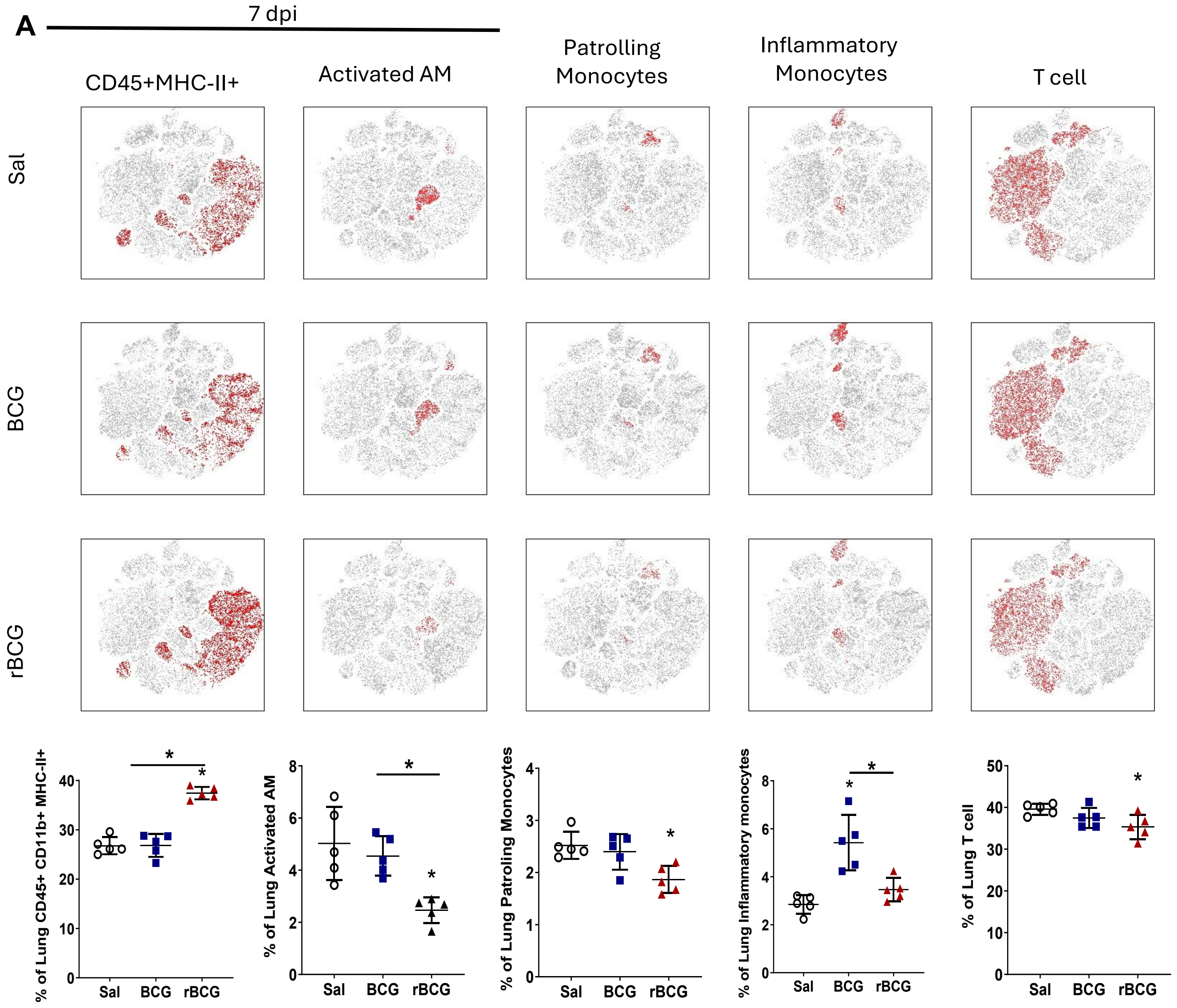

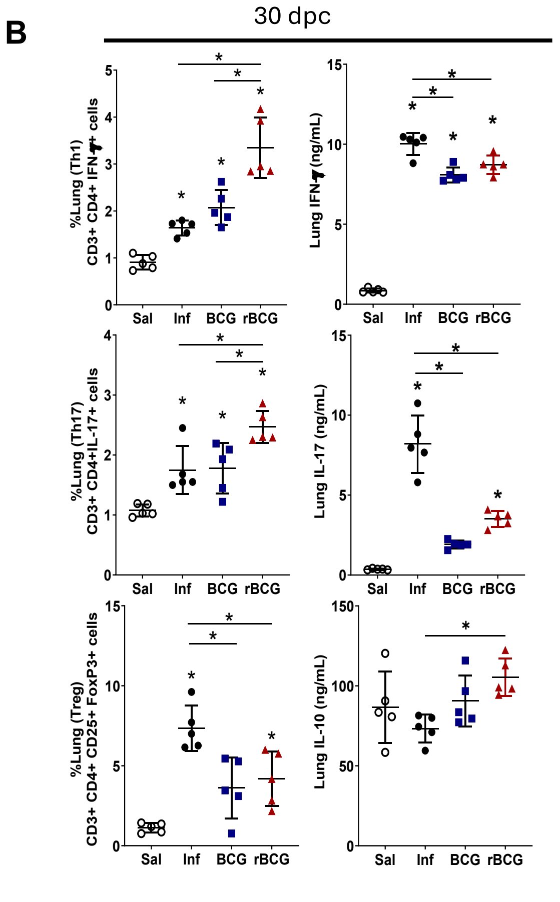

Supplementary Figure 4. Complete significant cytometry data. Lung tissue cells were evaluated by flow cytometry for effects of vaccination on cell population frequency. **(A)** At 7 dpi, lung cells were stained with antibodies to CD3, CD45, CD11b, CD11c, F4/80, Ly6C, Ly6G, MHC-II. Cells were characterized as bulk CD45+ MHC-II+ cells, activated alveolar macrophages (CD45+ CD11b+ F4/80+), patrolling monocytes (CD45+ CD11b+ Ly6C+), inflammatory monocytes (CD45+ CD11b+ CD11cHigh), and T lymphocytes (CD3+ CD45+) (n=5). **(B)** At 30 dpc, lung cells were stained for evaluating the presence of Th1, Th17 and Treg cell populations, using anti-CD45, CD3, CD4, IL-17, IFN-γ, CD25, and FoxP3 antibodies. Both individual data and mean ± SD are reported. Differences were considered statistically significant with P value < 0·05 in a one-way ANOVA test. An asterisk (*) above the group indicates statistical significance compared to Sal (control), while an asterisk with a bar indicates differences between groups.

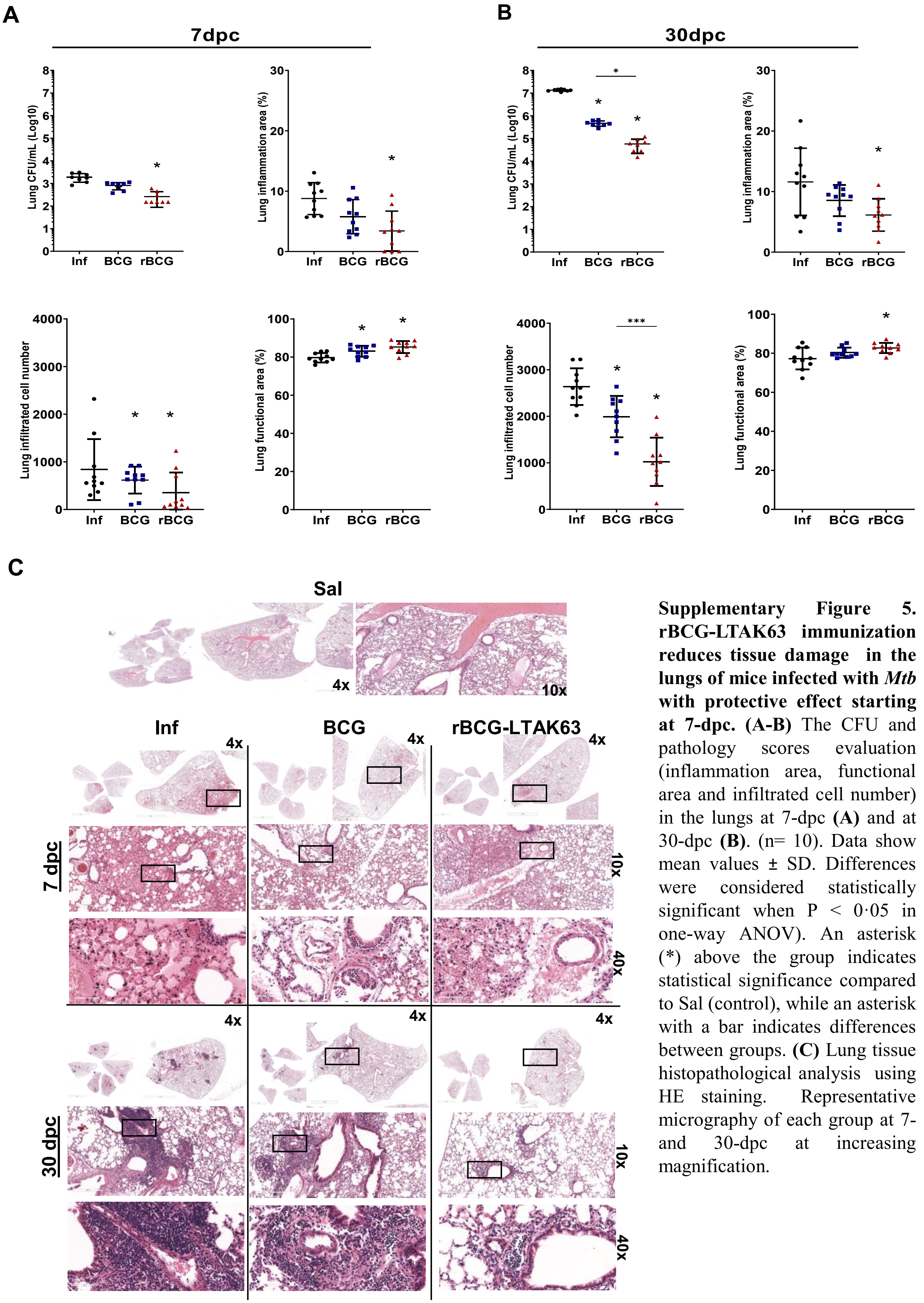

Supplementary Figure 5. rBCG-LTAK63 immunization reduces tissue damage in the lungs of mice infected with *Mtb* with protective effect starting at 7-dpc. (A-B) The CFU and pathology scores evaluation (inflammation area, functional area and infiltrated cell number) in the lungs at 7-dpc **(A)** and at 30-dpc **(B)**. (n= 10). Data show mean values ± SD. Differences were considered statistically significant when P < 0·05 in one-way ANOV). An asterisk (*) above the group indicates statistical significance compared to Sal (control), while an asterisk with a bar indicates differences between groups. **(C)** Lung tissue histopathological analysis using HE staining. Representative micrography of each group at 7- and 30-dpc at increasing magnification.

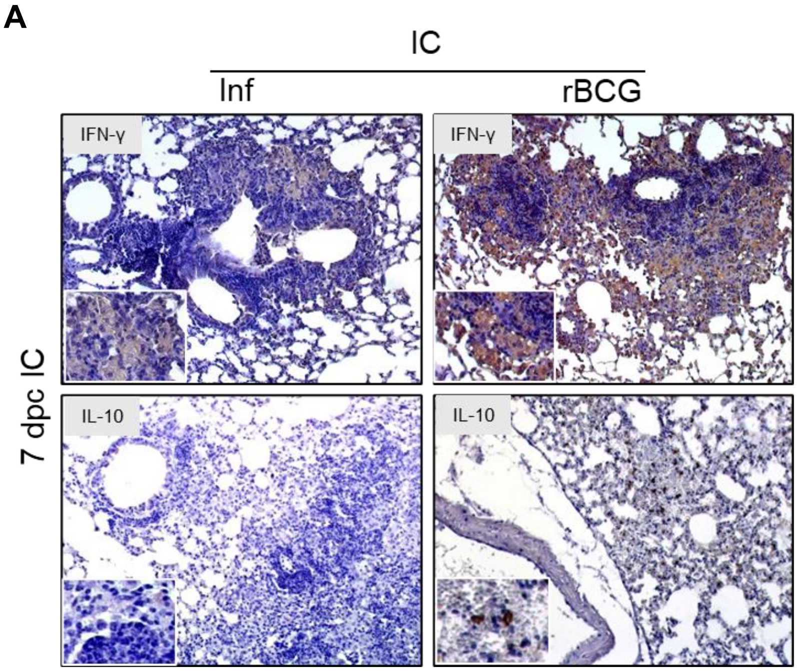

Supplementary Figure 6. Immunization with rBCG-LTAK63 enhances early IFN-γ responses and regulates transcriptional modules linked to pathological processes. (a) Sections of the same mouse lungs stained with anti-IFN-γ and anti-IL-10. Image shows the differences in lung infiltrated cells at 7 dpc . (b) Complete gene module enrichment analysis was performed on our dataset, to assess the impact of BCG or rBCG-LTAK63 immunization on modules associated with disease severity (susceptibility, infection dose, or strain virulence) (37,38) . Titles on the left side denote the functions associated to modules. Colors correspond to the average fold change relative to the Saline group. Only modules with specific functions and showing statistically significant differences.

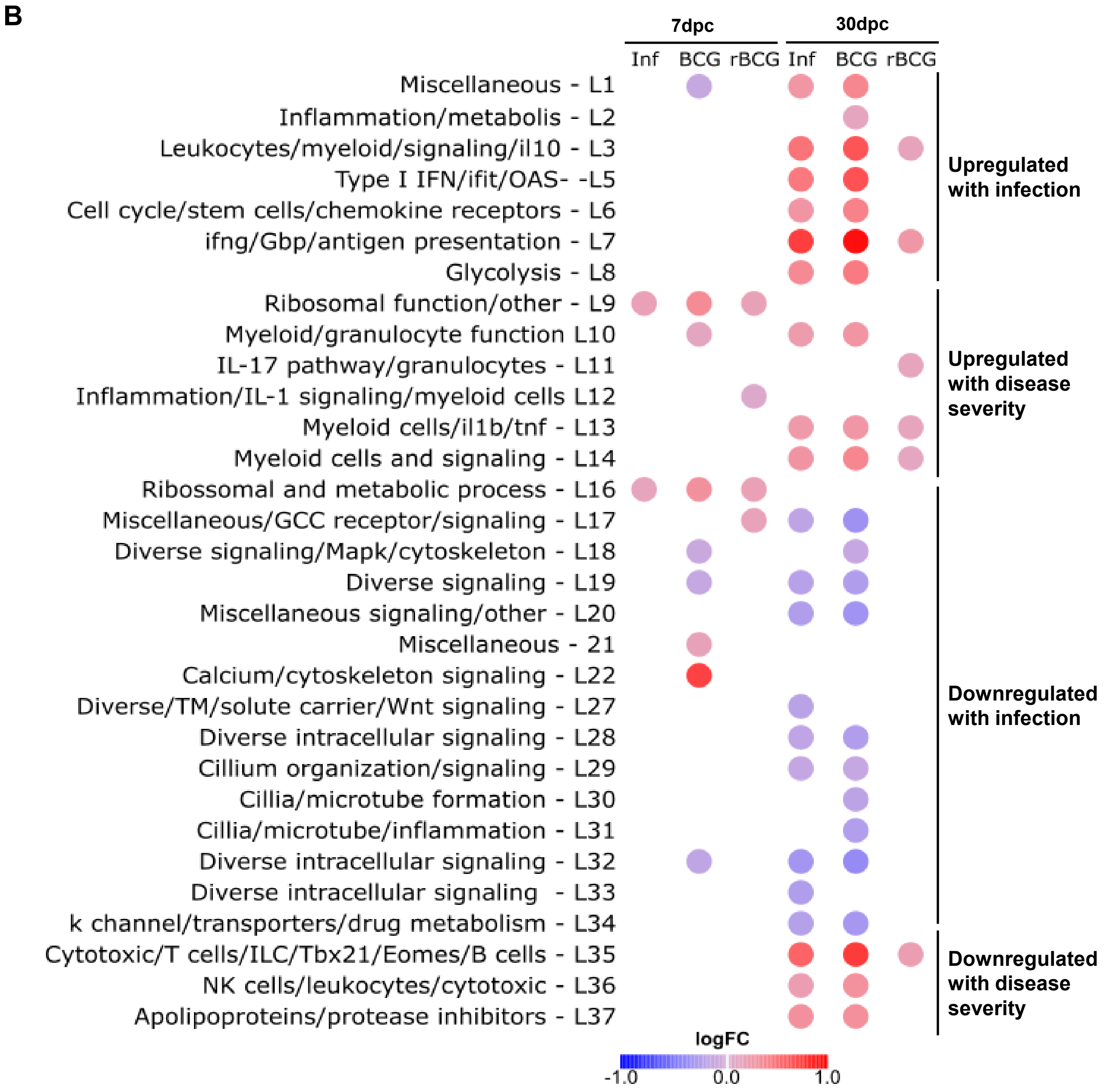

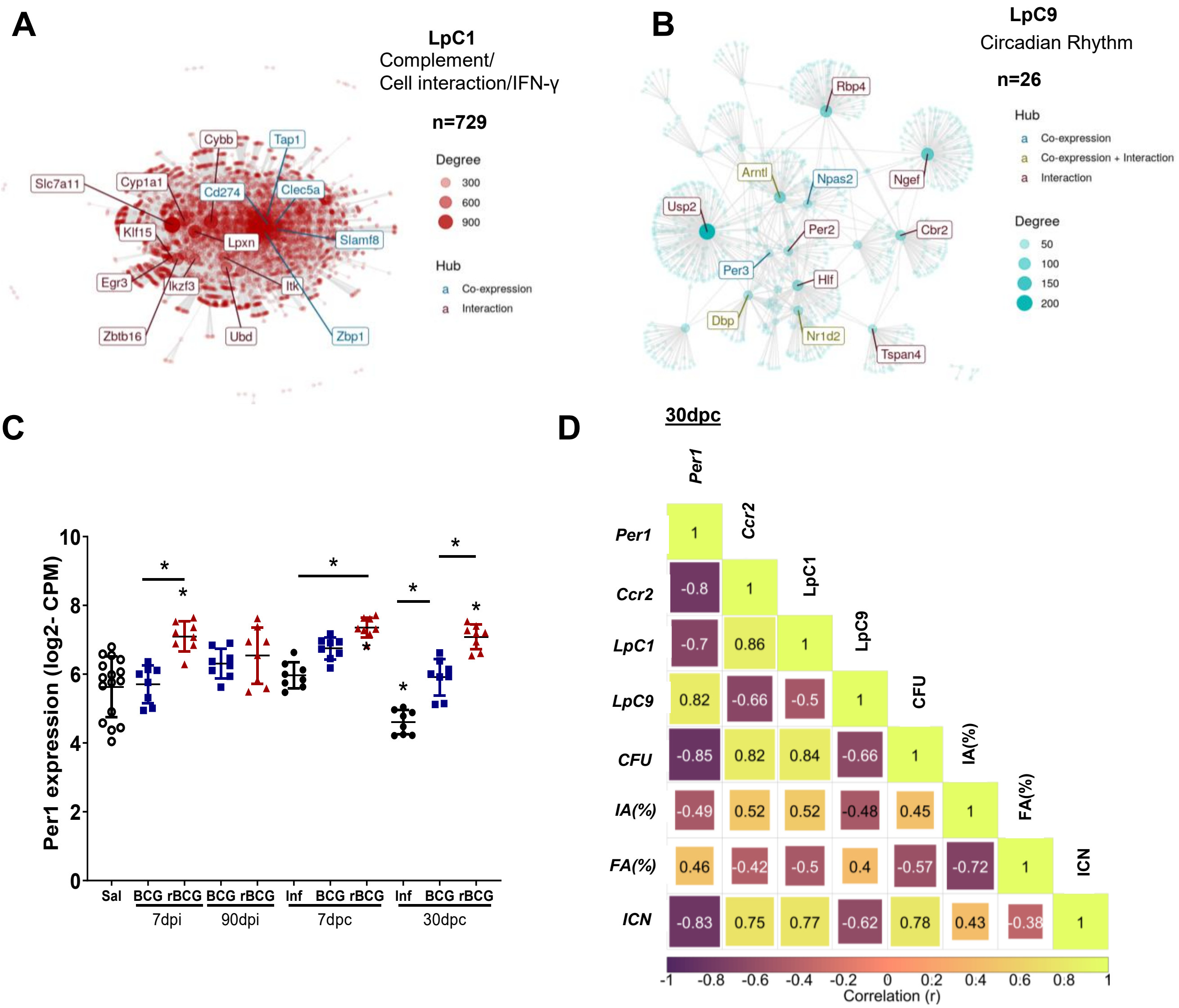

	Per1	Ccr2	LpC1	LpC9	CFU	IA(%)	FA(%)	ICN
Per1	1							
Ccr2	-0.8	1						
LpC1	-0.7	0.86	1					
LpC9	0.82	-0.66	-0.5	1				
CFU	-0.85	0.82	0.84	-0.66	1			
IA(%)	-0.49	0.52	0.52	-0.48	0.45	1		
FA(%)	0.46	-0.42	-0.5	0.4	-0.57	-0.72	1	
ICN	-0.83	0.75	0.77	-0.62	0.78	0.43	-0.38	1

Supplementary Figure 7. Co-expression analysis indicates that rBCG-LTAK63 modulates *Mtb*-induced inflammatory responses via the circadian rhythm pathway. Integrated network displaying interaction data for modules identified in the groups post-challenge. The number of genes identified in each module (n) is indicated below the module title. Interaction map displaying the connection between genes in **(A)** LcP1 and **(B)** LcP9. The nodes correspond to the 729 and 26 genes of modules, respectively, as well as the additional genes identified through interaction analysis. The genes are linked by co-expression and/or interaction. Gene network of modules for the most connected genes (hubs) are labeled and colored based on their "origin": if originally present in the CEMiTool module, they are colored blue; genes inserted from the interactions are colored red; gene identified in both are colored green. The magnitude of the node is directly proportional to its degree. **(C)** Per1 expression obtained from RNAseq (Log2 – CPM). Differences were considered statistically significant when P value < 0·05 in DeSEq2. The * above the group indicates statistical significance compared to Sal (control), while an * with a bar indicates differences between other groups. **(D)** Correlogram of Per1, Ccr2, eingengenes of LpC1 and LpC9 modules, CFU, inflammation area (IA), functional area (FA) and infiltrated cell number (ICN). Plot displays in purple colors the score negative value and yellow the score positive value of Pearson's/Spearman's correlation between the data, at 30dpc.

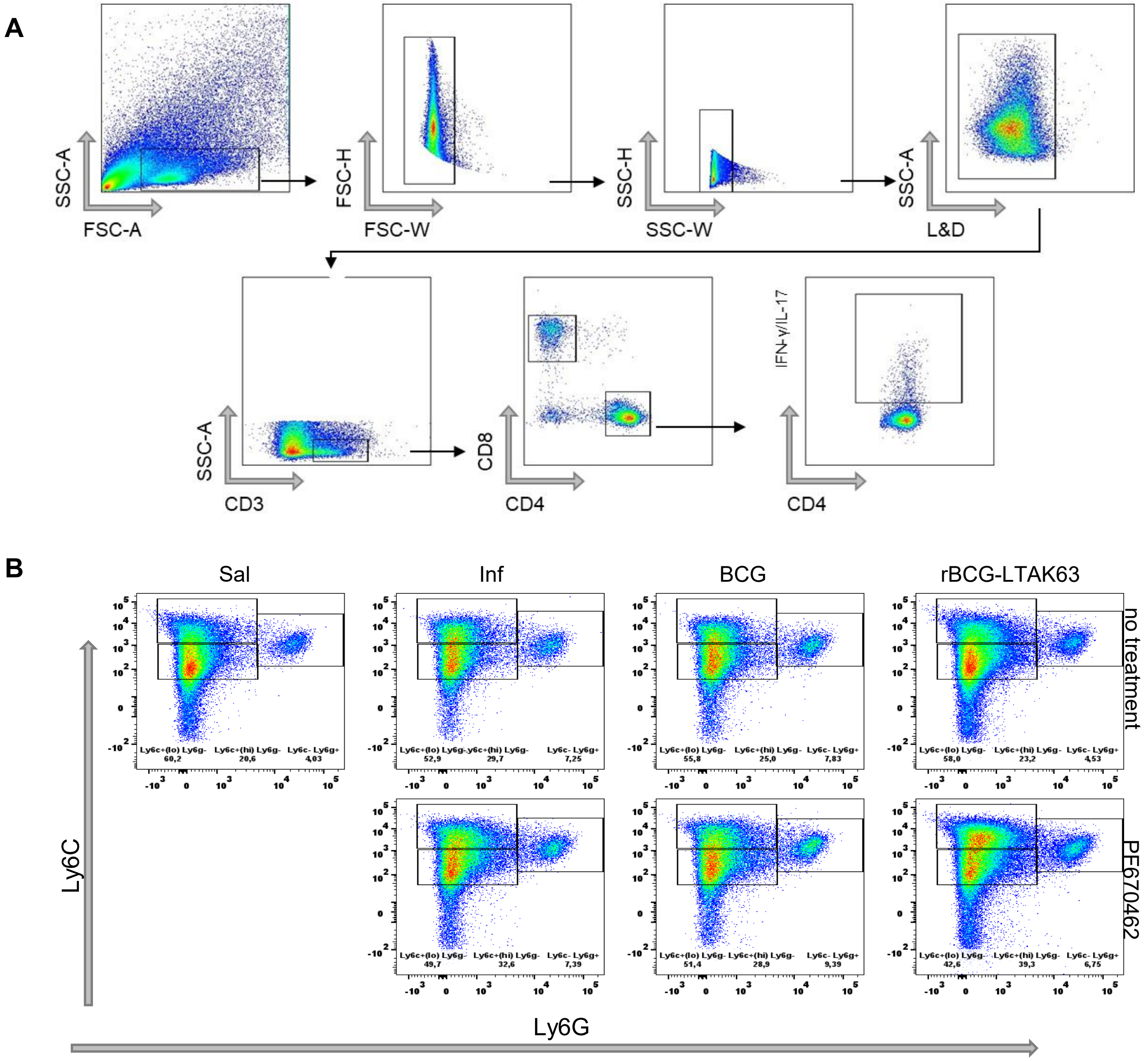

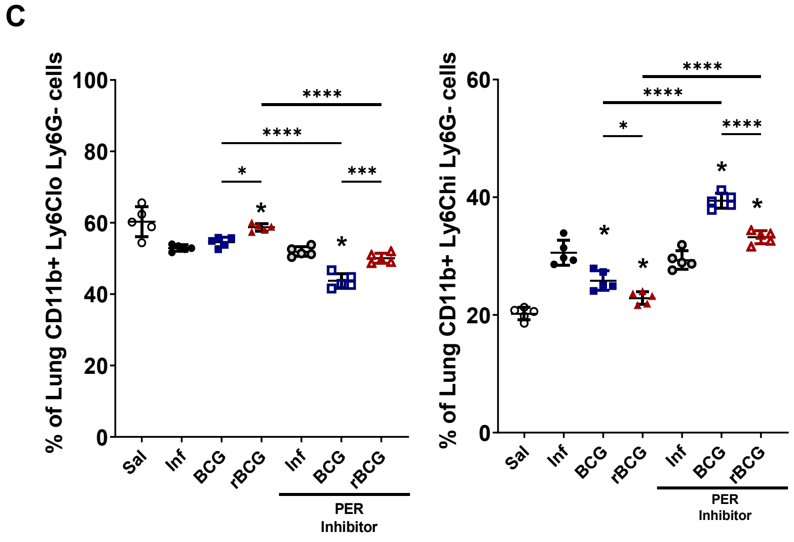

Supplementary Figure 8. Representative FACS plots showing the gating strategy of T cell expressing IFN-γ or IL-17 cells and monocytes and neutrophil populations across the groups. (A) Representative flow cytometry plots from lungs showing the gating scheme analyzing singlets, CD3+ cells, CD4+ CD8-, CD4+IFN-γ+ and CD4+IL-17+ cells, at 90-dpi. **(B)** Representative flow cytometry dotplot of Ly6C and Ly6G cells in the lungs of animals immunized or not with BCG or rBCG-LTAK63, treated or not with PF670462 and infected with *Mtb* H37Rv.

(C) Cells population frequency of patrolling monocytes (CD11b+ Ly6Clo Ly6G-), inflammatory monocytes (CD11b+ Ly6Chi Ly6G-), quantified by flow cytometry. Data is presented as mean ± SD. An asterisk (*) above a group indicates a significant difference compared to the Inf (control) group (ANOVA (*P < 0·05; ** P < 00·1; *** P < 000·1, and **** P < 0000·1), one-way ANOVA). An asterisk (*) with a horizontal bar denotes a significant difference between the indicated groups.
